# Fibrinogen *α*C-region acts as a functional safety latch: Implications for a fibrin biomechanical behaviour model

**DOI:** 10.1016/j.actbio.2024.10.005

**Published:** 2024-10-10

**Authors:** Tímea Feller, Helen R. McPherson, Simon D. Connell, Robert A.S. Ariëns

**Affiliations:** a Discovery and Translational Science Department, Leeds Institute of Cardiovascular and Metabolic Medicine, University of Leeds, UK; b Molecular and Nanoscale Physics Group, School of Physics, University of Leeds, UK

**Keywords:** Fibrinogen, Biomechanical model, Blood clotting, Polymer biology

## Abstract

Fibrin has unique biomechanical properties which are essential for its role as a scaffold for blood clots. Fibrin is highly extensible and demonstrates significant strain stiffening behaviour, which is essential for stress-distribution in the network. Yet the exact structures of fibrin at the sub-fibre level that contribute to its unique biomechanical characteristic are unknown. Here we show how truncations of the fibrinogen *α*C-region impact the biomechanical properties of fibrin fibres. Surprisingly, absence of the complete *α*C-region did not influence the low strain modulus of fibrin fibres but led to premature fibre rupture and decreased extensibility. Intermediate effects were observed with partial deletion of the *α*C-region, reflected by intermediate rupture stress and toughness. However, overall strain-stiffening behaviour remained even in absence of the *α*C-region, indicating that strain stiffening is not due to stress being transferred from the *α*C-region to the protofibril backbone. Upon stress-relaxation, decay constants and their relative contribution to the total relaxation remained similar at all strains, showing that a distinct relaxation process is present until fibre rupture. However, relative contribution of fast relaxation was maximal only in crosslinked fibres if the flexible *α*C-connector was present. These data show that the *α*C-region is not the main load-bearing structure within fibrin fibres and point to a critical role for the protofibril backbone instead. We present a revised structural model based on protofibril branching that fully explains the unique biomechanical behaviour of fibrin fibres, while the *α*C-region primarily acts as a safety latch at the highest of strains.

## 1 Introduction

Fibrin fibres have large extensibility [1], but the structures responsible for its remarkable biomechanical behaviour, such as the molecular arrangements of the inner structure of fibrin fibres, are still largely unknown. Molecular sub-structures of fibrin fibres are ~0.5 μm, which is the length of ~10 monomers [2,3], and aggregate laterally to form fibres. However, the length of these protofibrils within the formed fibres and how they may be connected longitudinally is unknown. As the internal architecture is a controlling factor of the remarkable mechanical properties of fibrin, it is of key importance.

protofibrils, which are double-stranded, half staggered assemblies of fibrin monomers. Protofibrils typically grow to the length of Prior art emphasized the role of structural elements in fibre integrity to be within the protofibril backbone, such as the unfolding of the coiled-coil region [4–7] or the *γ*-nodule in the D-region [8]. However, if all protofibrils were directly connected via their back-bone all along a fibrin fibre, this would result in a much lower extensibility of fibres and substantially higher fibre stiffness, comparable to the GPa range of stiffness of hard *α*-keratin fibres in which all *α*-helices are stretched [9], instead of the MPa range described for fibrin [10–13]. In an attempt to explain this reduced stiffness of fibrin fibres as well as their unusually large extensibility and non-linear elasticity, recent studies have suggested that besides these enthalpic unfolding events, enthropic behaviour is also present for fibrin [11,14,15]. This would mean that before stiffening of fibrin fibres, energy is invested in the decrease of entropy by alignment (straightening) of randomly coiled, unstructured region(s) [15]. As fibrin’s backbone is highly structured (folded), whereas the *α*C-region is not [16,17], the *α*C-region was consequently thought to define low-strain behaviour. The enthalpic process, meaning the unfolding of folded regions, only follows once the entropic alignment is complete. As unfolding events require larger invested energy, stiffness increases, resulting in strain-stiffening upon transition from entropic to enthalpic regimes. For fibrin, the unfolding of the *γ*-nodule [18], the coiled-coil region [7] or the *α*C-domain could all contribute to the enthalpic response. This model of entropic-enthalpic transition could provide an explanation for the low fibre stiffness at strains *<*100 % as the unstructured *α*C-region would require lower forces to unfold. It could also explain the strain stiffening response, because stress would get transferred to the protofibrils backbone from the *α*C-region, as well as the large extensibility, since the long *α*C-region would add to the total extensibility of all the connected protofibrils [11,14]. However, importantly, for this to be true, protofibrils would need to be connected not only laterally but also longitudinally via the *α*C-region [14,15], which would significantly limit protofibril length.

Modifying or deleting parts of the fibrin monomer, the building blocks of the fibrin fibre, using recombinant technology can elucidate the role of these structures. Prior art indicates that the length of the *α*C-region correlates with extensibility. The shorter *α*C-region in chicken fibrin resulted in reduced extensibility when compared to human [19]. Yet, as it is fibrin from different species, part of the decrease may be due to alteration in other structures, like in the structure of the A and B knobs [20]. In support of a role for the *α*C-region in fibre extensibility, a recent study showed increased extensibility for a recombinant fibrin variant based on a rare human natural variant with an extended *α*C-region (*α*EC) [21]. So far there are no studies on how gradual shortening of the *α*C-region in human fibrinogen affects mechanical behaviour at the fibre level, despite its clinical significance: the fibrin(ogen) *α*C-region is the first structural element in fibrin that undergoes proteolytic degradation *in vivo* [22–24]. Additionally, both FXIII crosslinking sites and *α*2-antiplasmin binding sites are located in the *α*C-connector region, making fibrin more resistant to fibrinolysis [25]. Consequently, we previously showed how loss of the *α*C-region increases fibrinolysis and decrease clot stability [26]. This is of particular importance in traumatic injuries where fibrinolytic activation is common, leading to the excessive truncation of the *α*C-region, correlating with poor clinical outcome [27] and excessive blood loss [28]. In agreement with this, mutations leading to truncation in the *α*C-connector region in patients are associated with a bleeding phenotype [29].

Our work presented here is the first experimental work to investigate the role of the *α*C-region in the mechanical behaviour of human fibrin fibres by selectively truncating either the *α*C-domain (*α*390) or the complete *α*C-region (*α*220). Based on the currently available model for protofibril and *α*C-region arrangements, we expected the *α*C-region to be a main load-bearing structure in fibrin fibres at low strains, as recent models predict that protofibrils are mechanically and structurally coupled through the *α*C-regions [14,15]. However, here we show that even the complete absence of the *α*C-region in *α*220 results in a largely similar mechanical behaviour of fibrin fibres, particularly at low (<150 %) strains.

Thus, our data lead us to propose a new, structural model of the internal structures of fibrin fibres that does not require the *α*C-region to be the main load-bearing structure at any strains. We propose that branched protofibrils [30] form random connections within fibrin fibres. This allows selective loading of limited numbers of protofibrils. Meanwhile, the *α*C-region acts as a safety latch at higher strains, bringing together unstrained protofibrils and increasing their probability to engage with binding sites that have been released due to unfolding of the strained protofibrils, thus supporting further extension prior to rupture. This new model for protofibril arrangements within fibrin fibres is able to fully explain the biomechanical behaviour of these unique natural fibres that underpin important blood clot characteristics in health and disease.

## 2 Materials and methods

### 2.1 Fibrinogen

Recombinant wild type (WT) and truncated *α*-chain variants of fibrinogen were expressed in Chinese hamster ovary (CHO) cells and purified by affinity chromatography using the calcium-dependent IF-1 antibody [31]. For further details on fibrinogen expression and purification, please see McPherson et al. [26]. No changes were made in the *β*- and *γ*-chains. Three variants were used in this study: 1) WT, with full length *α*C-region, and a normal *α*-chain of 66 kDa, 2) *α*390, in which the *α*C-region was truncated after aspartate 390, resulting in the loss of the *α*C-domain and a reduction of the *α*-chain molecular weight to 42 kDa, and 3) *α*220, in which the *α*C-region was truncated after serine 220, further reducing the *α*-chain molecular weight to 25 kDa and resulting in the loss of the complete *α*C-region, including both the *α*C-domain and the flexible *α*C-connector region. Protein homogeneity and fibrin crosslinking was checked with sodium dodecyl sulphate polyacrylamide gel electrophoresis (SDS-PAGE) (Fig. S1/A). Protein (2 μg) of each recombinant variant was loaded on a Nu-PAGE Bis–Tris gel (Thermo Fisher Scientific) after denaturation. The gel was run at 160V and then stained with InstantBlue (Abcam, Cambridge, UK).

### 2.2 Experimental methods

**Fig. 1:**
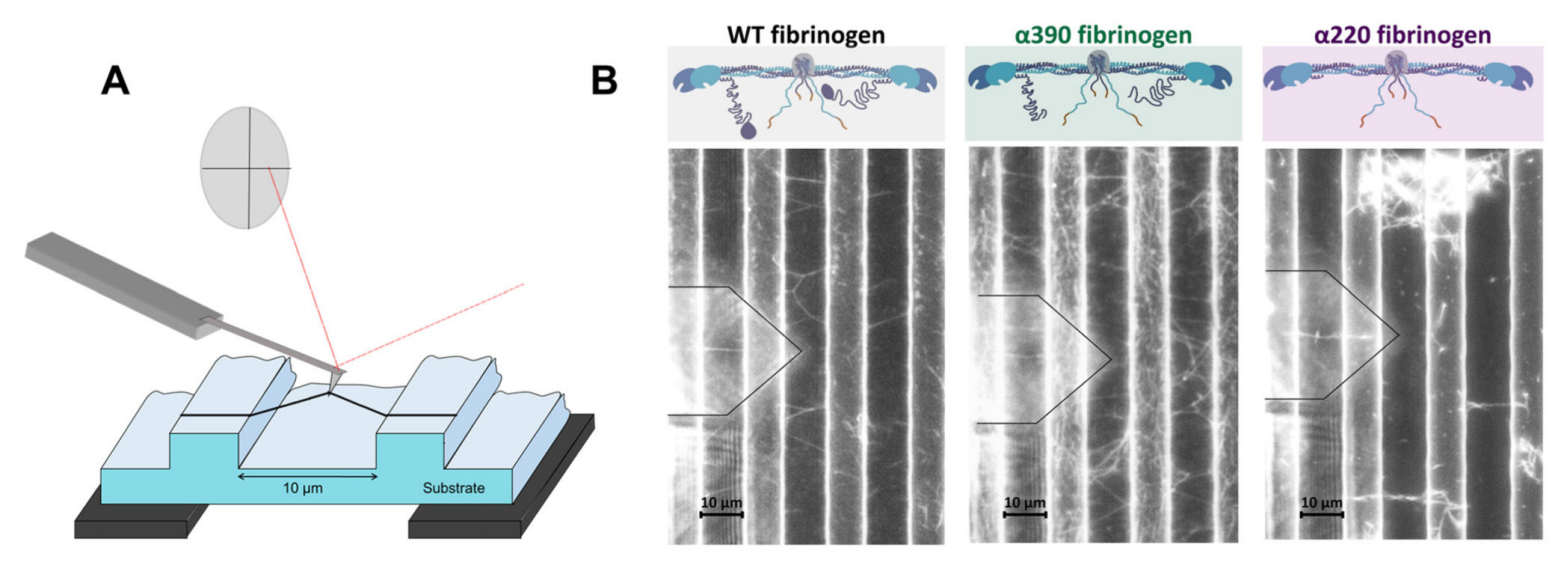
Lateral fibre pulling and fibrinogen variants used. A) Fibrin fibres were pulled in lateral direction with the cantilever of an atomic force microscope (AFM). The lateral deflection and the Y-movement of the cantilever was used to calculate the stress-strain behaviour of the fibre (Fig. 2/A). B) 3 variants of recombinant human fibrinogen were used, either with intact *α*C-region (WT), with the globular *α*C-domain truncated but the flexible *α*C-connector remaining (*α*390, green), or with the complete *α*C-region removed (*α*220, purple). Molecular models of each variant are above the representative fluorescent microscopy images of the network they formed on the striated surface. The shadow of the cantilever can be seen on the left side of each image, outlined with black.

An activation mix containing thrombin and calcium was added to a fibrinogen solution (fibrinogen, with/without FXIII), and 20 μl of this mixture was immediately placed on a striated substrate. Final concentrations were: 0.5 mg/ml recombinant human fibrinogen, 0.1 IU/ml thrombin (human, Sigma-Aldrich), 15 μg/ml FXIII (Zedira, Darmstadt, Germany) 5 mM calcium in TRIS-buffered saline (TBS; 50 mM TRIS, 100 mM NaCl, pH 7.4). Samples were left to clot for 2 h in a humidified chamber at room temperature, after which excess clot and fibrin film [32] were removed using a pipette. Samples were gently washed with TBS-buffer and then labelled with 20-nm-yellow-green carboxylate FluoSpheres (Thermo Fisher Scientific) for 10 min, then gently washed again. Fibre pulling measurements were carried out in buffer, using an atomic force microscope (AFM) (MFP3D-BIO, Asylum Research) equipped with an epifluorescence microscope (Axiovert 200, Zeiss). Data were collected and parameters calculated as described before [33]. Briefly, the sample was placed so that the wells and ridges of the striated surface run perpendicular to the length of the AFM cantilever. Fibres reaching over wells of the striated substrate, parallel with the length of the cantilever and perpendicular to the ridges, were pulled with the AFM cantilever (HQ:CSC38/NoAl, μMasch) in a direction lateral to the cantilever’s length (Fig. 1/A). The pulling process was followed using fluorescence microscopy. Stress and strain were calculated (see Theory/Calculation section below). Fibres were excluded from the analysis if they slipped on the ridge before rupture or connected with any additional branch to any other point on the ridge, or if they ruptured before stiffening. Mechanical properties of the fibres were tested by pulling them with a speed of 0.1 μm/s in the following three different manners:

### 2.3 Single fibre pulling with continuous speed until rupture

**Fig. 2:**
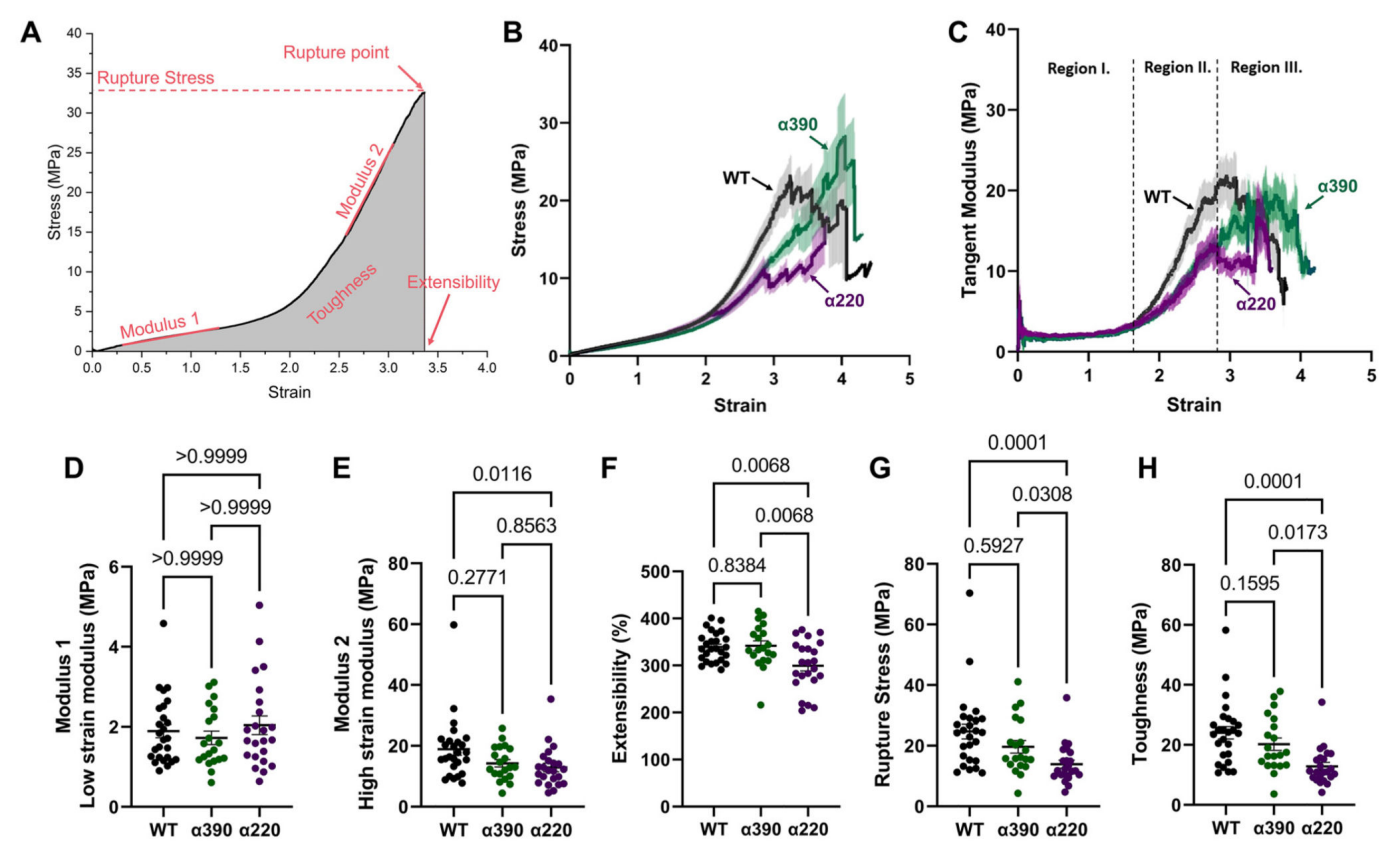
Mechanical behaviour of fibrin fibres: single fibre pulling. A) Stress-strain behaviour of fibres. Parameters marked with red were measured on each fiber’s stress-strain curve. B) Row statistics ± SEM stress-strain curves of all single fibre pulls for each variants (crosslinked, WT = 26, *α*390 = 20 and *α*220 = 23). Row statistic curves show the average ± SEM of all fibres measured for each variant at each strain increment. Note that above strains of 2, fibres start to rupture, dropping out of the row statistics curve. C) Row statistics ± SEM curves of the tangent moduli for each variant. Tangent moduli were calculated by derivation of the respective stress-strain curves. Adjacent averaging was used with a window of 50 data points. Resulting curves were used for row statistics. D–H) parameters were measured as shown on Fig. 2/A. *P*-values are represented above the data.

The first type of experiment was to pull at single fibres with constant speed until rupture. The following parameters were measured from the stress-vs-strain curves (Fig. 2/A): *Rupture Stress* and *Extensibility*, the maximal stress and strain the fibre ruptured at, respectively. *Toughness* is the area under the stress-strain curve, which reflects the total energy the fibre can bear before rupture. Fibre stiffness was characterised by *Modulus 1*, the initial slope of the fibre at low (<1.5) strains and *Modulus 2*, the stiffness the fibre reaches after stiffening, before rupture. To characterise fibre stiffness at all strains, tangent modulus was calculated as the first derivative of the stress-strain curve and plotted against strain after adjacent averaging of 50 points with the sampling resolution of 10 points/s. To minimize day-to-day errors, repeated measurements were done including measurements of all variants we wished to compare on each day (either crosslinked (X) or uncrosslinked (UX)). As it was not possible to measure more than 4 sample type (variants, X or UX) on the same day, uncrosslinked fibres were measured and tested separately from crosslinked fibres, with WT crosslinked re-measured as a control. Due to day-to-day variability, we did not directly compare the crosslinked and non-crosslinked data.

### 2.4 Incremental strain-steps pulling of single fibres

**Fig. 3:**
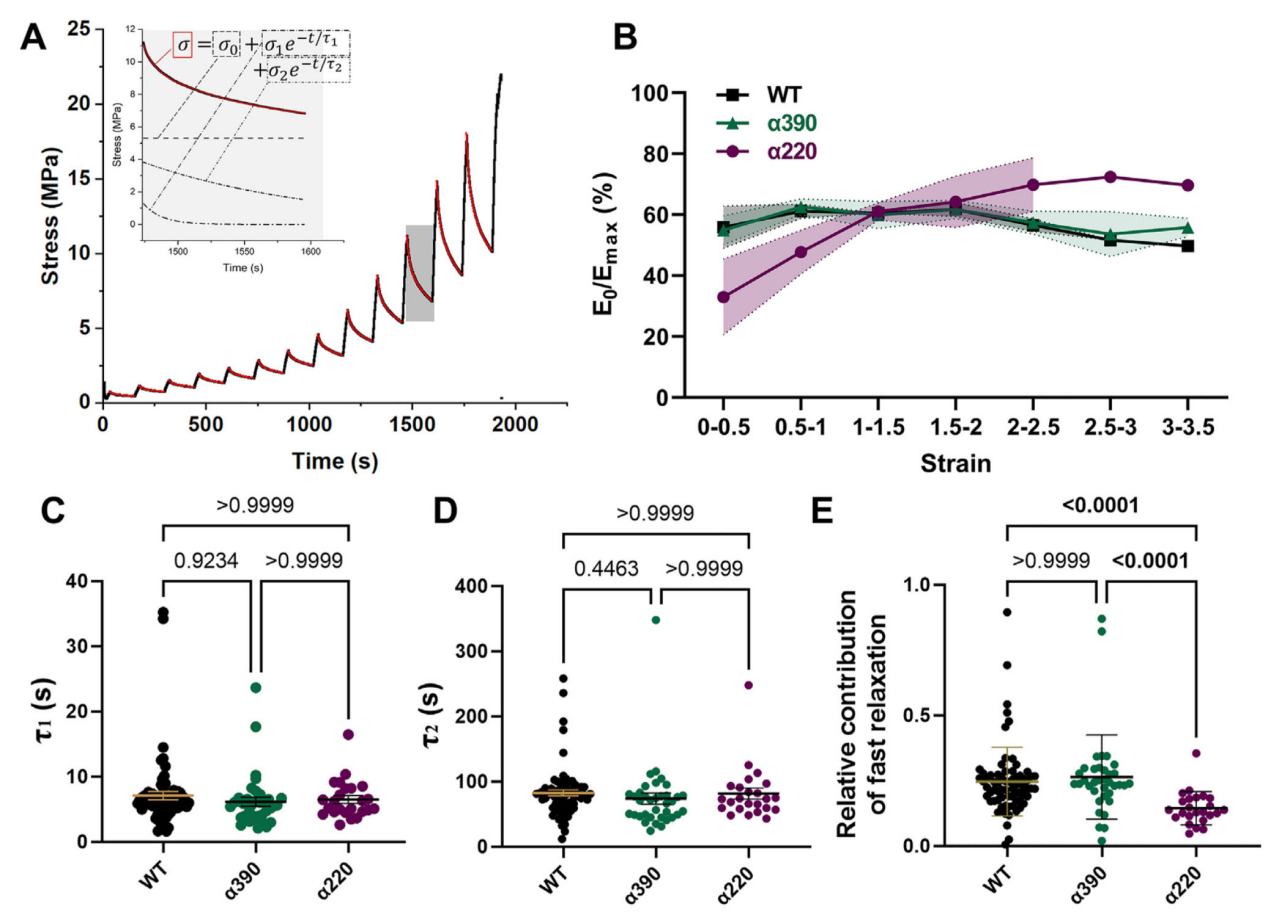
Mechanical behaviour of fibrin fibres: Incremental fibre pulling of crosslinked fibres. A) During incremental fibre pulling, fibres are left to relax after each 2 μm strain-increment. The exponential decay during the relaxation was characterised by using the ExpDecay2 function of Origin. Fitted curves are represented with red colour. Inset with grey background visualises the 3 functions: *σ*_0_, present at all timepoints like a baseline, $\sigma_{1}\cdot e^{-t/\tau_{1}}$ representing fast relaxation and $\sigma_{1}\cdot e^{-t/\tau_{2}}$ representing slow relaxation. For all measured curves see Fig. S4/A,B. B) elastic proportion of the mechanical behaviour, calculated as *E*_0_/*E*_max_ (%) = *σ*_0_/*σ*_max_ (%). All curves represented here are average ± SEM (dashed line and shadow) of values within strain increments of 0.5. For raw data see Fig. S4/C. C) Comparison of fast decay constants *τ*_1_ for each crosslinked variant. No strain-dependence was observed for *τ*_1_ (Fig. S4/D,E) thus data at all strains were grouped and compared. D) Comparison of slow decay constant *τ*_2_ for each crosslinked variant. Note the difference in Y-axis compared with *τ*_1_. No strain-dependence was observed for *τ*_2_ (Fig. S4/D,F) thus data at all strains were grouped and compared. E) Relative contribution of the fast elastic decay to all decay processes, calculated as *E*_1_/(*E*_1_ + E_2_). Similar to *τ*_1_ and *τ*_2_, we found no strain-dependence in the Relative contribution of the fast elastic decay (Fig. S4/G) thus data at all strains were grouped and compared.

The second type of experiment involved pulling at single fibres in steps, allowing fibres to relax in-between the sequential pulls before pulling again. After each 2 μm increase in the cantilever’s Y position, fibres were left to relax for 2 min. During these relaxation times, stress decayed in a double exponential manner, as described previously [10]. We used the ExpDecay2 function of Origin for characterization. Viscoelastic properties of fibrin fibres were investigated from the fitting parameters (Fig. 3/A inset). The offset of the decay, *σ*_0_ is where the curve would have decreased if left for infinite time and thus shows the relaxed stress, the purely elastic proportion of the behaviour. Meanwhile, the stress lost during the decay (*σ*_max_-*σ*_0_) is indicative of viscous processes. Thus, the elastic proportion of the mechanical behaviour was calculated as *σ*_0_/*σ*_max_, which is equal to the ratio of the elastic modulus vs. total modulus *E*_0_/*E*_max_, as both *σ*_0_ and *σ*_max_ were measured at the same strain. Similar to the fibre pulling until rupture, we could not compare the crosslinked with the uncrosslinked dataset due to day-to-day variability. However, some parameters were independent of the actual stress values (which are most prone to error), and thus such parameters could be compared between the sets of experiments. These were decay constants *τ*_1_ and *τ*_2_, as well as any ratios like the relative contribution of fast relaxation, which therefore could be compared between the two sets of experiments.

### 2.5 Cyclic fibre pulling of single fibres

To test fibre recovery and measure permanent deformation, we tested fibrin fibres by pulling them repeatedly 3 times to strains 1, then 3 times to strain 2 and 3 times to strain 3, with a speed of 0.1 μm/s and with no delay prior to return. We chose these strains to characterise distinct regions of the mechanical response (see Section 3.1). After each pull, we returned to zero strain. Previous work suggested that labelling fibrin fibres with fluorescent microbead inhibits their lysis [34] and leads to a preferred tendency of fibres to elongate instead of getting cleaved upon lysis [35]. It opens the possibility of structural rearrangement of solid beads during fibre extension that could then hinder the elastic recovery, leading to permanent deformation. Consequently, we decided to use Alexa488 labelled fibrinogen instead of the fluorescent microbeads for the cyclic fibre pulling. We labelled each variant using Alexa Fluor 488 using a protein labelling kit (Thermo Fisher Scientific). Labelling efficiency was 0.82, 0.73 and 1.65 moles dye per mole protein for WT, *α*390 and *α*220 variants, respectively. Clots were made by adding 20 % of this labelled fibrinogen.

We sometimes experienced a slight lateral drift of the cantilever that varied between 0.015–0.293 V, while the lateral signal of the pulling was in the 2.26–8.02 V range. As the drift is slight compared with the lateral signal of the fibre pulling it doesn’t alter the previous results but would impact the measured permanent deformation of the cyclic pulling. Thus, we determined the drift by finding a baseline of the Lateral deflection vs. Time curves using the Baseline treatment function of Peak Analyzer in Origin, then subtracted the baseline from the lateral signal. Stress and strain were then calculated as described in Section 2.6.

### 2.6 Theory/calculation

The pulled fibre deflects the cantilever in the lateral direction *(L*_*v*_ [V]), and this lateral deflection was used to calculate the force experienced by the fibre *(F*_*fibre*_ [N]) during the pulling process:

$$F_{fibre\;}=\frac{L_{v}\times k_{e}}{2*\text{sin}(\alpha )}$$

where *α* is the angle between the fiber’s original and current position while *k*_*e*_ [N/V] is the effective spring constant of the cantilever, estimated by careful calibration before each day’s experiment and calculated after Liu et al. [10]. In detail:

$$k_{e}=k_{l}\times S_{l}=\frac{G\times w\times t^{3}}{3\times l\times (h+t/2)}\times \frac{E\times (h+t/2)\times InvOLS}{2\times G\times l}$$

where *E* is the elastic moduli of silicon (G, the viscous moduli of silicon cancels out from the equation). *w, h* and *l* are the width, height and length of the cantilever. InvOLS is the inverse optical lever sensitivity [m/V], which was measured as the slope of the contact region of *DeflInvOLS* [V] vs. *distance* [m] curves in liquid medium, by pressing the cantilever against glass surface. The thickness of the cantilever *t* was calculated from the spring constant of the cantilever *kn*, (obtained from the Thermal graph of the cantilever in air) as follows: $\sqrt[{3}]{k_{n}\times 4\times l^{3}/(E\times w)}$.

After the fibre had ruptured, the fiber’s remaining parts on the ridge were scanned in contact mode and the image was used to measure the fibre height *(h)* and width *(w)*. Stress experienced by the fibre (*σ* [Pa]) at all strains was calculated from $F_{fiber\;}[\text{N}]:\sigma_{fibre\;}=\frac{F_{fiber\;}}{A}$, using the cross-sectional area $A=h\times w\times \frac{\pi }{4}$ of the fibre (considering an ellipsoidal shape). Strain (*ε*) was calculated from the cantilever’s Y-position *(l’)*[μm]: $\varepsilon =\frac{l^{\prime }-5}{5}$ where 5 stands for the 5 μm half-width of the 10 μm wide wells.

### 2.7 Statistical methods

Crosslinked variants (WT, *α*390 and *α*220) were measured in 6 separate parallel runs, where all 3 types of samples (WT, *α*390 and *α*220) were measured in the same run, to minimize the effect of day-to-day differences (see details in Error estimation section, for uncrosslinked variants see Supplement Section S3). Numbers of measured fibres were: WT *X* = 26, *α*390 *X* = 20 and *α*220 *X* = 23. In order to choose the right statistical tests to compare variants, data distribution was tested with normality test. If any of the variants failed the normality test, we used nonparametric Kruskal–Wallis test for comparison. If all variants passed the normality test, we used ordinary one-way Anova. Consequently, we used the Kruskal–Wallis test when comparing Rupture Stress, Modulus 1 and Modulus 2, and Anova when comparing Extensibility and Toughness.

For row statistics, mean and SEM of stress values at each strain were calculated by averaging all fibres measured for each variant (X or UX) and plotted against strain.

For incremental pulling experiments, row statistics was not possible as the strain values, where fibres were left to relax, were not exactly the same. Consequently, resulting parameters of the exponential decays were grouped in 0.5 strain increments for better visualisation and analysis. Note that double exponential fitting has some uncertainty, especially at strains <0.5 due to the changes in stress being close to the limit of sensitivity of the cantilever.

### 2.8 Error estimation

There are two main factors which may result in error of measurement. One is the calibration of the cantilever and consequently the estimation of its spring constant, and the other is the measurement of fibre cross-sectional area. The error of the calibration of the cantilever gives fairly similar results for the lateral spring constant thus is less considerable. The other factor is the measurement of the height and width of the fibre, which incurs uncertainty for the following reasons: the same cantilever is used for fibre pulling as for scanning, thus contamination of the cantilever is possible, resulting in an increase of the tip radius and overestimation of the fibre width. Additionally, the cantilever ideal for fibre pulling is relatively soft, thus less ideal for imaging. There can also be subtle differences between different batches of cantilevers in terms of size and shape.

These errors were not present for the calculations of the following parameters: 1) Extensibility, where neither fibre radius, nor the cantilever’s spring constant is needed for the calculation. 2) decay constant of the incremental pulls 3) any ratios, where these errors cancel out. This includes the stiffening ratio, as well as the elastic proportion for incremental pulls. Consequently, we included all variants (both crosslinked and uncrosslinked) in the analysis of the incremental pulls, but treated the crosslinked and uncrosslinked datasets of continuous pulls separately.

Further to the above-mentioned errors, the shape of the tip may artificially broaden fibres on the AFM image. This was accounted for by measuring filament width at half height for all fibres [36]. We used engineering stress for our calculations as unfortunately we can’t measure diameter of suspended fibres during pulling in these experiments and thus, thinning of the fibres at large strains can’t be accounted for. Consequently, our high-strain stiffness is likely underestimated, but this underestimation is similar for all variants, making them comparable. Slipping of fibres on the ridge during pulling would increase the error of extensibility, but as slipping can be seen on the fluorescence microscopy image and these fibres were excluded from analysis.

We do not expect mechanical behaviour to be altered upon fibre pulling by the presence of fluorescent microbeads as they are on the fibre surface, and we don’t change the direction of pulling. However, for cyclic fibre pulling, where presence of the microbeads might result in permanent deformation (see Section 2.5), we chose to use Alexa488 labelling so that the permanent deformation of each cycle was measurable. As fibre pulling is stopped to test re-laxation in incremental pulls, we tested if labelling with fluorescent microbeads alters fibre relaxation. No difference was found upon labeling with fluorescent microbeads (see Fig. S2).

### 2.9 Fibre pre-selection

Removing the excess clot and fibrin film (Section 2.2) is an essential step experimentally so that fibres above the wells can be manipulated. But it also opens the possibility of pre-selection of fibres. We would like to emphasize that only fibres well attached to the ridges of the substrate can be measured, otherwise the fibres would slip during the pulling. This attachment will also protect these fibres from detaching while the excess clot is removed. Thus, this pre-selection of fibres by the removal of the excess clot is experimentally beneficial.

We only used striated substrates with a 10 μm well width. Although this is experimentally necessary so that the variants are comparable on fibre and sub-fibre level, it introduces a pre-selection of the fibres as only fibres longer than 10 μm were measured. At the same time, if measuring fibres in a network, a wide variety of fibre length and width is included.

## 3 Results

### 3.1 Single fibre pulling and rupture behaviour

We first studied crosslinked fibrin fibres as our previous work had shown that complete truncation of the *α*C-region resulted in unmeasurably low elastic moduli in the absence of crosslinking at the local network level when measured with a magnetic micro-rheometer, due to substantial changes in clot network structure [26]. Once clots were prepared on the striated surface for lateral pulling of single fibre (Fig. 1/A), we confirmed structural differences between WT, *α*390 and *α*220 variants (representative examples in Fig. 1/B), consistent with our previous report [26]. Truncation of the globular *α*C domain (*α*390) resulted in a denser network of thinner fibres, while truncation of the complete *α*C-region (*α*220) resulted in clumps of varying density with stunted fibres and large pores. Despite these structural alterations of the fibre network, for both truncations fibres could be found that fully stretched between the ridges of the micro-grid. In contrast with the differences in network appearance, the radii of fibres that could be pulled were largely similar, apart from crosslinked *α*220 fibres that had marginally greater radii than WT fibres (Fig. S1/B,C). This difference in apparent structure from fibre to network level is because our experiments are necessarily limited to fibres longer than 10 μm and can’t include shorter and thinner fibres (see Section 2.9).

When crosslinked single fibres of each variant were tested by lateral pulling, the overall mechanical behaviour was surprisingly similar between all three variants (Fig. 2), with the stress-strain curves closely overlapping at low strains (Fig. 2/B), and strain stiffening (the increase of modulus 2 compared with modulus 1, Fig. 2/A) was present for all variants. We next calculated the derivative of stress-strain curves to obtain the tangent modulus, representing fibre stiffness at all strains (Fig. 2/C). For these tangent modulus curves, 3 distinct regions were apparent. First, tangent moduli were identical at strains up to ~1.5 (Fig. 2/C, Region I), and we found no difference in the low-strain stiffness (modulus 1), neither for *α*390 nor for *α*220 when compared with WT (*p >* 0.9999 for any groups, Fig. 2/D). Second, the mechanical behaviour of the variants became different only at larger strains in Region II: the tangent modulus started to diverge from WT above strains of ~1.8, but interestingly the tangent modulus of *α*390 and *α*220 still did not diverge from each other up until strains of ~2.8 (Fig. 2/C). The decreased amount of stiffening during Region II resulted in the *α*390 variant fibres plateauing at a lower tangent modulus in Region III, also reflected in the reduction of high-strain stiffness or modulus 2 (Fig. 2/E, *α*390 vs. WT 0.76-fold, *p* = 0.2771). Third, above strain of 2, fibres started to rupture. This region that is dominated by fibre rupture is defined as Region III (Fig. 3/C). Most of the rupture events take place above strains of ~2.8 (Fig. 2/F) but it is important to note that around half of the *α*220 fibres had already ruptured by strain of three, while most of the WT and *α*390 still remain intact. Extensibility of the *α*220 variant was significantly decreased, compared with both WT and *α*390 variants (0.882 and 0.874-fold, respectively, *p* = 0.0068 in both case), while there was no significant difference between the extensibility of WT and *α*390 variant (1.009-fold change, *p* = 0.8384). The early rupture of the *α*220 variant led to an even further reduction in maximal stiffness of the fibres before rupture: modulus 2 for *α*220 vs. WT was decreased 0.68-fold, *p* = 0.0116 (Fig. 2/E). Furthermore, step-wise removal of the *α*C-region resulted in gradual decrease in rupture stress (Fig. 2/G) and toughness (calculated as the area under the stress-strain curve; Fig. 2/H and Table 1).

Surprisingly, and in contrast with the local network behaviour in microrheology experiments [26], we found that the biomechanical properties of uncrosslinked individual fibres were not unmeasurable for all truncated fibrinogen variants. Similar to our previous findings [12], in the absence of crosslinking, WT fibres displayed lower strength at all strains and lower high strain moduli when compared with WT crosslinked fibres (Fig. S3) although these changes we measure are somewhat milder (Table S1). This was applicable for all uncrosslinked variants when compared with crosslinked WT (see supplement, section S3, Fig. S3). In the absence of the *α*C-region (*α*220), fibre extensibility reduced by a similar degree for uncrosslinked as for crosslinked fibres (Table 1). The reduction in toughness and rupture stress was somewhat milder upon *α*C-truncation for the uncrosslinked fibres as compared with the crosslinked variants (Table 1).

### 3.2 Incremental pulls and viscoelastic properties

Next, we investigated the mechanical behaviour of crosslinked fibrin fibres with consecutive pulling interspersed with periods of time for the fibres to relax. We found that double exponential decay equations fitted the relaxation of fibrin best (Fig. 3/A), with the two defined decay constants being well separated. By fitting double exponential decay, all relaxations were decomposed into an offset and 2 curves: *σ*_0_, present at all timepoints like a baseline, a fast relaxation $\sigma_{1}\cdot e^{-t/\tau_{1}}$ and a slow relaxation $\sigma_{2}\cdot e^{-t/\tau_{2}}$ (Fig. 3/A inset). Raw data of all incremental pulls and acquired fit parameters are represented in Fig. S4. Taking into account the strain (*ε*) the decay was measured at, stress values *σ*_max_, *σ*_0_, *σ*_1_ and *σ*_2_ can be converted into elastic moduli *σ* = *ε* · *E*, as follows:

$$\varepsilon \cdot E_{\text{max}}=\varepsilon \cdot \left[E_{0}+E_{1}\cdot e^{-t/\tau_{1}}+E_{2}\cdot e^{-t/\tau_{2}}\right]$$

Stress relaxation is indicative of viscous processes [10,37] which represent structural rearrangements, while offset *σ*_0_ is purely elastic. Consequently, by calculating the ratio of the relaxed and total (initial) stress *σ*_0_/*σ*_max_ (%), which is equal to the ratio of the relaxed and total modulus *E*_0_/*E*_max_ (%), we can characterise the purely elastic proportion of the mechanical behaviour. We calculated *E*_0_/*E*_max_ (%), and for better visualisation grouped them in strain increments of 0.5 (Fig. 3B). *E*_0_/*E*_max_ (%) appeared some-what strain-dependent and increased at strains <1, reaching a peak at medium strains (*ε* = 1–1.5), and then tended to decrease again at larger strains (Fig. 3/B), indicating some increase in structural rearrangements at larger strains. The large variability of *E*_0_/*E*_max_ (%) at low (<0.5) strains is possibly due to protofibril [15] or fibre straightening, a deformation dissipating energy. However, most importantly, stress decay was present at all strains for each crosslinked variant. This indicates that structural reorganisations occur at all strains, and not only at large strains. Additionally, fibre relaxation is quite prominent: including all relaxations measured here, *E*_0_/*E*_max_ (%) turned out to be 56.8 % (±0.708 % SEM) on average, indicating that 43 % of the total stress (and total modulus) is partially used for remodelling and structural reorganisation within the fibre.

We also investigated the time constant of both relaxations, representing the characteristic time in which the initial stress decreases to 1/e (~37 %). Fast and slow relaxation regimes separated well at all strains with the slow relaxation constant *τ*_2_ being *>*10 times the fast relaxation constant *τ*_1_ (90.4s ± 3.02 vs. 7.3s ± 0.23 SEM respectively, including all measured data). As neither of the decay constants showed strain dependence, we compared variants by using all measured decay constant at any strains. We found no significant difference in *τ*_1_ or *τ*_2_ between any of the truncated variants (Fig. 3/C,D). Importantly, both *τ*_1_ and *τ*_2_ were present in all truncated variants. Consequently, neither of the relaxations can be directly assigned to the *α*C-region.

To investigate if either the fast or the slow relaxation became more prominent in the partial or complete absence of the *α*C-region, we calculated the contribution of the fast relaxation to the total relaxation using *E*_1_/(*E*_1_ + *E*_2_) and called it relative contribution of fast relaxation. The lower this value is, the smaller proportion the fast relaxation contributes to the total relaxation. This parameter didn’t show strain dependence either, indicating that none of the relaxation processes gains importance over the other with increasing strains. However, when we compared variants with each other, a significant decrease was measured for only the *α*220 variant (Fig. 3/E). This indicates that the fast relaxation is impaired in the absence of the complete *α*C-region (*α*220), but not in the absence of the globular *α*C-region alone (*α*390). Decreased prominence of fast relaxation in the absence of the *α*C-region is also indicated when it’s initial amplitude, *E*_1_ was compared to the initial modulus *E*_max_ at the start of relaxation (Fig. S5/B). This means that even though the characteristic fast relaxation time remains, it’s initial amplitude, *σ*_1_ and consequently its prominence is decreased when the *α*C-region is not present. Loss of the *α*C-region did not change the contribution of slow relaxation (Fig. S5/C). For further details on the strain dependence of *τ*_1_, *τ*_2_ and relative contribution of fast relaxation please see supplement Fig. S4/E–G.

In the absence of crosslinking, a double exponential decay was also present and the strain dependence of *E*_0_/*E*_max_ % was similar to the crosslinked variants (Fig. S5/A). No significant differences were found in *E*_0_/*E*_max_ (%) in the absence of crosslinking or upon *α*C-region truncation (Fig. S5/D). For each variant, both *τ*_1_ and *τ*_2_ was higher in the absence of crosslinking (Fig. S5/E,F, *p <* 0.05; with the exception of *τ*_1_ for *α*220). The absence of crosslinking also significantly decreased relative contribution of fast relaxation for both WT and *α*390 variants (Fig. S5/G, Table S5). However, for the *α*220 variant, where the relative contribution of fast relaxation was already reduced, the absence of crosslinking did not decrease this parameter any further. This shows that if either the *α*C-region is missing or crosslinking is absent, fast relaxation loses its prominence but still clearly remains.

Relaxation is indicative of viscous processes, but not a purely viscous behaviour. To calculate both the elastic and viscous moduli of relaxation we used the generalized Kelvin model utilizing Maxwell elements (Fig. S6) [38]. There the viscous moduli (μ) is calculated as μ = E•*τ* [10]. Even though moduli changed somewhat with increasing strains as shown in Fig. 3/B, we still found significant decreases in all moduli of relaxed and fast relaxation (*E*_0_, *E*_1_, *μ*_1_) when comparing both crosslinked variants (*α*390 and *α*220) to WT (Fig. S6). Although we see a similar tendency, no significant changes were shown between any uncrosslinked variants.

### 3.3 Cyclic fibre pulling

**Fig. 4:**
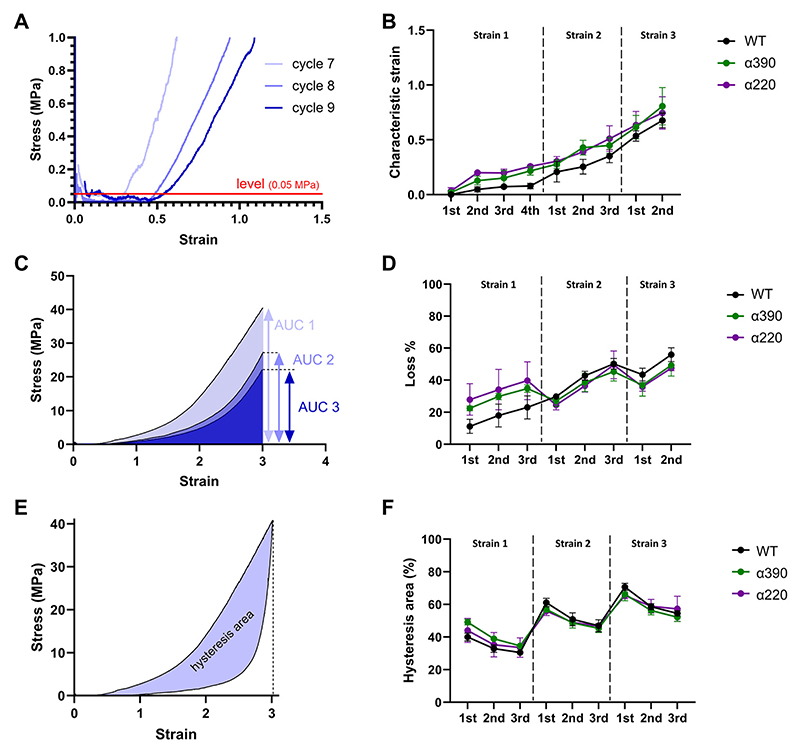
Mechanical behaviour of fibrin fibres: Cyclic fibre pulling. A) Characteristic strain is the strain where the pulling curve of each cycle crossed a given stress level. We chose this level to be 0.05MPa B) Characteristic strain increases in consecutive cycles, indicating permanent elongation. Average ± SEM is represented C) Loss% was measured by relating the area under the pulling curve to the area under the first pull at each strain-step: AUC_2_/AUC_1_ × 100 % or AUC_3_/AUC_1_ × 100 %. D) Loss% indicates permanent deformation. Loss% increased the most between the first and last cycle for the WT variant. Average ± SEM is represented E) Hysteresis area% was calculated by relating the area of the hysteresis between the pull and release curve to the area under the pull curve ((AUC_pull_ -AUC_release_)/AUC_pull_ × 100 %). F) Within each strain increment, hysteresis decreased in consecutive cycles. Average ± SEM is represented.

Three fibres of each variant were repeatedly pulled three times at each of three incrementally increasing strains, namely strains 1, 2 and 3, then released to strain 0 at the end of each cycle. To avoid any possible permanent deformation due to the presence of the fluorescent microbeads, we labelled these samples with Alexa488 (see Section 2.5). Stress-strain curves are represented in Supplement Fig. S7. All three variants seem to show similar stress-strain behaviour and no clear difference can be seen. The following parameters were measured and compared. 1) Characteristic strain. We measured this parameter similar to earlier work [21], by estimating the strain where the stress reached a level that is distinguishable from background fluctuations. This level was 0.05 MPa here (Fig. 4/A). Characteristic strain indicates permanent elongation of fibres at the end of each cycle. Importantly, characteristic strain increased gradually with increasing maximal fibre extension and in cycles with strain of 3 it reached a permanent elongation up to 0.8–1 (Fig. 4/B). This clearly shows that at high strains a significant proportion of the extension is due to a permanent structural reorganization. 2) Loss%. This parameter shows how much energy is lost upon repeated pulling at each strain-step. The area under the pulling curve was related to the area under the first pull at the given strain-step (AUC_2_/AUC_1_ × 100 % or AUC_3_/AUC_1_ × 100 %) (Fig. 4/C). This parameter shows permanent deformation, but besides fibre elongation it is also impacted by further structural rearrangements within the fibre. Loss% reaches a maximum at high strains indicating that permanent structural deformation is the most significant at high strains, as might be expected. An important observation is the increase of loss% with increasing strains is most prominent for the WT variant (increasing from ~10 % to ~55 %), indicating that the presence of the *α*C-region increases the propensity for a permanent structural change at increasing strains. 3) Hysteresis area% was calculated as the difference between the area under the pull and release curve in each cycle and related to the area under the pulling curve ((AUC_pull_-AUC_release_)/AUC_pull_ × 100 %). For viscoelastic materials, hysteresis is indicative of viscous behaviour. At each strain step, hysteresis was highest at the first pull and decreased in consecutive pulls. As the return curves remained largely similar (Fig. S7), these decreases in the hysteresis area are largely due to the change in the pulling curve, indicating that some of the viscous behaviour is turned into permanent deformation following consecutive cycles, agreeing with the increase in Loss% described above.

## 4 Discussion

Our results show that the mechanical behaviour of fibrin fibres is mainly determined by structures other than the *α*C-region at most strains: 1) Absence of even the complete *α*C-region did not change the stress-strain behaviour at low strains, where it was thought to be largely responsible for the mechanical response. 2) Strain-stiffening still happened in the absence of the *α*C-region, and thus strain stiffening does not involve strain being transferred from the *α*C-region to the protofibril backbone. Additionally, relaxation constant *τ*_1_ and *τ*_2_, as well as the relative contribution of fast relaxation were strain independent, suggesting that the load bearing structure within fibrin fibres remained similar at all strains, both before and after strain stiffening. 3) No change was found in decay constants *τ*_1_ and *τ*_2_ upon the loss of the *α*C-region, showing that none of the decay regimes can be directly assigned to the *α*C-region 4) Even though the fast relaxation gets less prominent in the absence of the complete *α*C region as shown by the decreased relative contribution of fast relaxation, it is still present, making up for ~16 % of the total relaxation. 5) All variants showed largely similar behaviour upon cyclic pulling. All these data demonstrate that the key load-bearing structures within individual fibrin fibres remain largely similar in the absence of even the complete *α*C-region, and thus the *α*C-region is not the load-bearing backbone within individual fibrin fibres, especially at lower strains. However, the flexible *α*C-region does play a key role in fibres reaching their maximal extensibility, consequently their maximal rupture stress and toughness. Thus, the flexible *α*C-region acts like a safety latch, allowing fibres to extend even further and reach their maximal strength before final rupture. Our results highlight the need for a new, revised model of the intra-fibrillar protofibril arrangements that underpin fibrin fibre structure and function.

### 4.1 Structural model

#### 4.1.1 Protofibril backbone

If protofibrils were longitudinally connected via the *α*C-region, the mechanical response should be largely affected by truncation of the *α*C-region, either resulting in stiff, brittle structures with decreased extensibility if the entropic domains can hold the structure together, or an extremely weak material without structural integrity if not. However, we found no such effects of *α*C-truncation, showing that the energy required to stretch individual fibres at low strains involves structures other than the *α*C-region. If energy is used for stretching any part of the *α*C-region at these low strains, it is unmeasurably low compared with the energy required to stretch the structure which has the load-bearing role at these low strains.

**Fig. 5:**
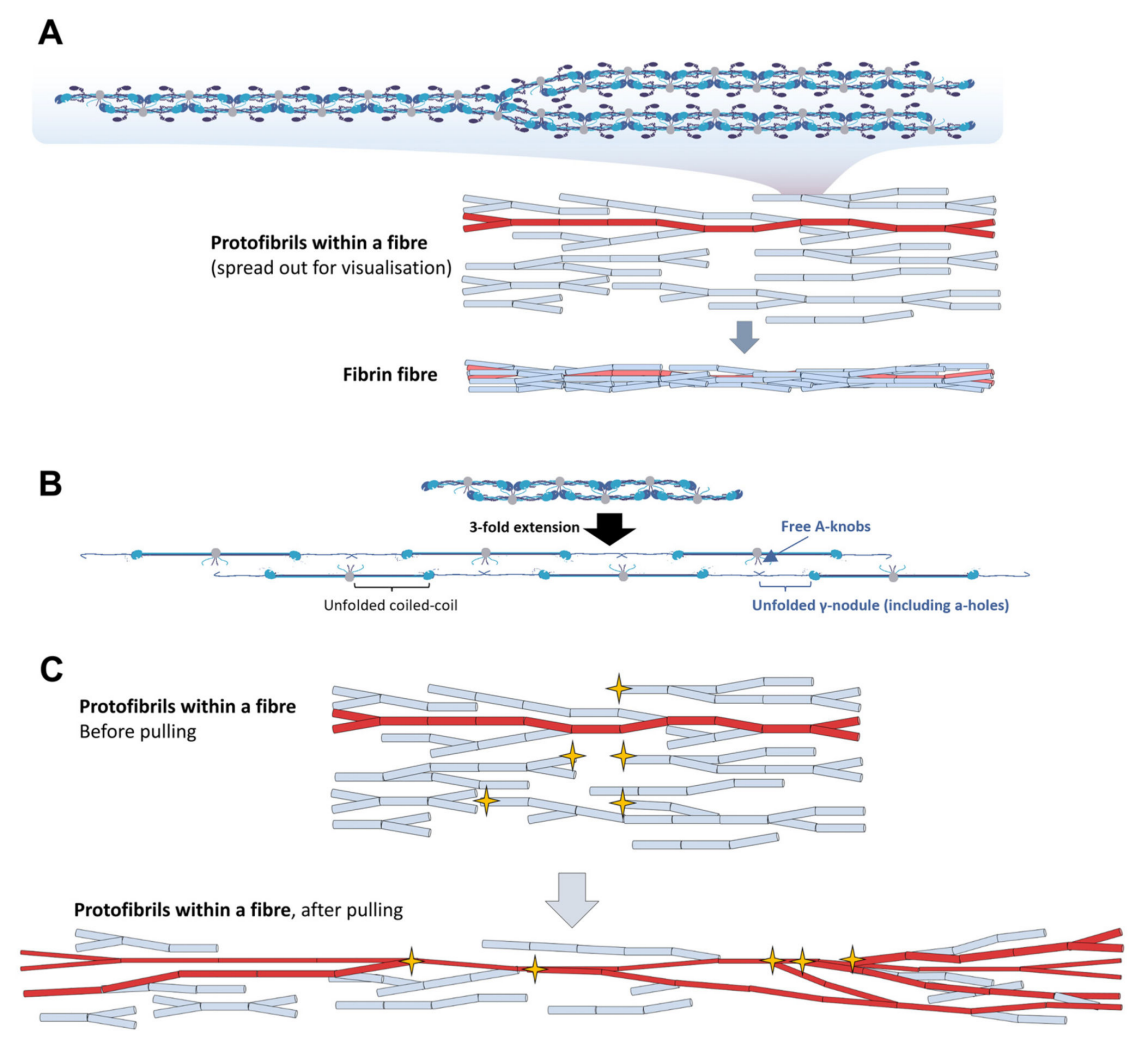
Organisation of protofibrils within fibrin fibres: New model of selective protofibril loading underpinning fibre extensibility and strain-stiffening. A) Protofibrils are branching structures that collapse into a fibre upon network formation. Each rod represents a protofibril ~10/10 monomers. Protofibril(s) long enough to reach all along the fibre are represented with red colour. These bear the load upon stretching, while stunted protofibrils, marked with blue, remain initially unloaded. B) Upon strain of 2 (3-fold extension), both the coiled coil and the *γ*-nodule unfolds, leaving free knobs all along the protofibril. C) As the fibre gets loaded, free holes at the stunted ends of so far unloaded protofibrils (marked with yellow stars) may bind to the excess of free knobs of the unfolded, selectively loaded strand. The increased number of protofibrils bearing the load leads to strain stiffening of the fibre.

The protofibril backbone has to be responsible for the stress-strain behaviour of fibrin fibres when the *α*C-region does not contribute significantly. Prior art also emphasizes the importance of the protofibril backbone, showing the key role of knob-hole interactions in fibrin polymerization. The *α*C-region, although it facilitates polymerization [26,39,40], is not sufficient on its own to lead to fibrin polymerization [33]. Yet, as mentioned before, a continuous protofibril backbone would result in much stiffer fibres. Additionally, strain-stiffening cannot be explained with such a structure. Thus, we propose that protofibrils along the fibrin fibre are not continuous but randomly connected via branching points. Frequent branching of protofibrils has been described previously [30], but not previously included in any model of the inner structure of fibrin fibres. We propose that during the rapid assembly of polymerizing fibrin, random connections form between protofibrils, resulting in only a few protofibrils that are long enough to stretch the full length of the fibre (Fig. 5/A, red) while most of the protofibrils are not long enough to stretch the full fibre and end stunted (Fig. 5/A, blue). In such a structure, protofibrils will be selectively loaded once a fibre is pulled: the short, stunted protofibrils remain unloaded while protofibrils stretching the full length of the fibre will bear the mechanical load. Consequently, the low stiffness of the fibre at low strains is due to the low number of protofibrils being stretched. The backbone of the loaded protofibrils, namely the coiled-coil and the *γ*-nodule starts to unfold (Fig. 5/B). Unfolding both the coiled-coil and the *γ*-nodule can accommodate strain up to 2 [14], which can cover extensions up until the start of the strain stiffening. Notably, with *γ*-nodule unfolding, the holes of knob-hole interactions get disrupted within the protofibrils. Consequently, the two branches of the loaded protofibrils become detached, and multiple knobs become available for new interactions.

Holes of other, so far unloaded, adjacent protofibrils may now bind these newly available knobs. Due to these new protofibrils joining in to the main, loaded protofibril strand, the number of loaded protofibrils increases (Fig. 5/C), leading to the strain-stiffening of the fibre. The catch-slip nature of knob-hole interactions [41] may further help to stabilize these newly formed connections [42]. All along the stretching process, protofibrils can slide relative to each other. Consequently, fibre extensibility is not limited by the maximal extensibility of the protofibrils around strain of 2. Notably, as the joining in of the adjacent protofibrils is highly variable and random (just as the longitudinal organisation of protofibrils in this model), the extensibility will largely vary from fibre to fibre. Similar to our results here (Fig. 2/F), high variability in fibre extensibility was described before [10,15,43], but previous models could not provide an explanation for this variability. Reorganization of protofibrils based on newly generated knobhole interactions during fibre pulling would suggest permanent deformation of the fibres, especially at strains<2 which was clearly indicated by our cyclic pulling experiments.

#### 4.1.2 Role of the αC-region

**Fig. 6:**
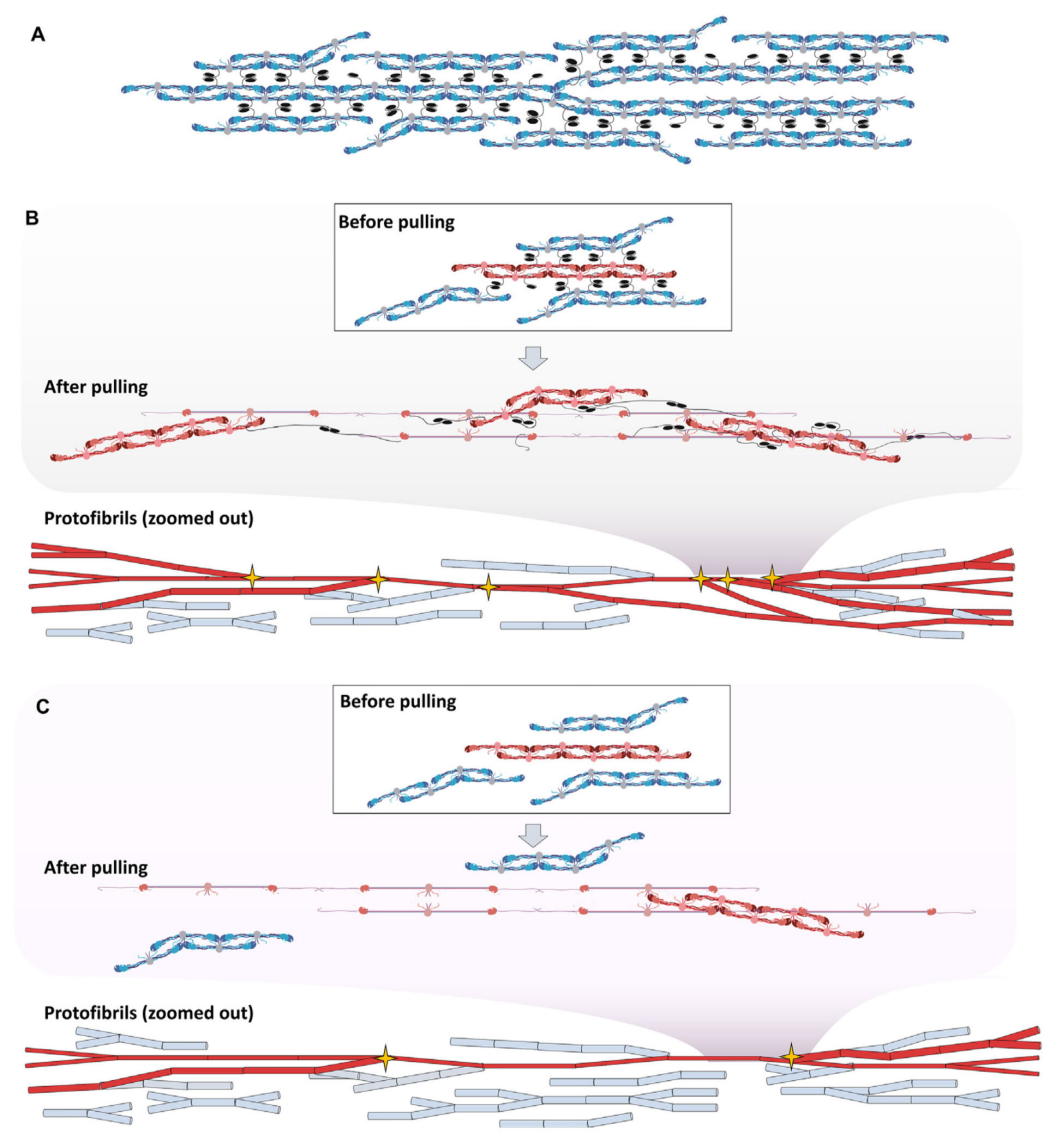
Role of the *α*C-region during fibre extension. A) In our revised model, protofibrils are connected only laterally via the *α*C-region. B) Extension of protofibrils in the presence of the *α*C-region in our model. Load-bearing protofibrils are marked with red, non load-bearing protofibrils marked with blue. The selectively loaded protofibril (red) extends and slides as gamma nodules unfold. The flexible *α*C-region pulls adjacent protofibrils closer to the main, selectively loaded protofibril thus increasing the probability of new knob-hole bond formation. As a result, more protofibrils contribute to the high-strain behaviour and reinforce the structure. C) Extension of protofibrils in the absence of the *α*C-region. Load-bearing protofibrils are marked with red, non load-bearing protofibrils marked with blue. The selectively loaded protofibril extends and slides as gamma nodules unfold, yet the *α*C-region is not present to pull adjacent protofibrils closer. Thus, the probability of knob-hole bond formation with adjacent protofibrils decreases. As a result, less protofibril will be available to reinforce the structure.

In the meantime, the *α*C-region does not possess a significant load bearing function. Yet, in its absence fibres tend to rupture prematurely. Similar to previous work [44,45], our model assumes that the *α*C-region connects protofibrils laterally but not longitudinally (Fig. 6/A). Up to strain of 2, the flexible *α*C-connector should accommodate the elongation of the adjacent, stressed protofibril. For this to be true, alterations are needed in the connection of adjacent protofibrils, as represented on Fig. 6/A: the maximal extension of the flexible *α*C-connectors is 50 nm [14] thus it can accommodate 200 nm extension of the protofibril backbone (4 × 50 nm, as 2 *α*C-connectors are attached both at the first and last connection point), allowing only the extension of 2 monomer length without the need of the globular *α*C-domain to unfold. Previous models were also limited by this length [14,15]. In our model protofibrils are only connected laterally via the *α*C-region and not longitudinally. Consequently, protofibrils are not limited in length by the extensibility of the *α*C-region: instead of ending, they diverge away from one another. Such divergences can be explained by the inherently bent conformation of the protofibrils [46]. Note that the energy required to straighten the unfolded *α*C-region is negligible compared with unfolding events within the protofibril backbone, thus no difference is expected in low-strain modulus in the absence of the *α*C-region.

Upon further extension, however, these lateral connections via the *α*C-region act like an anchor, and pull adjacent, so far untensed protofibrils closer to the selectively loaded protofibril (Fig. 6/B). This closeness increases the probability of the available holes of the unstrained protofibrils to bind to the newly freed-up knobs of the strained protofibril. In the absence of the *α*C-region, these knob-hole bonds can still form upon loading, but as the *α*C-region is not present to pull the protofibrils closer together, the probability of these interactions decreases substantially (Fig. 6/C). This leads to lower number of protofibrils bearing the load which results in lower fibre extensibility and consequently in the lower toughness we measured. Altogether, the *α*C-region acts as a safety latch, especially at high strains, and prevents the early rupture of fibrin fibres. Our results show the key importance of *α*C-connector region in this behaviour, as extensibility is only significantly reduced for the *α*220 and not for the *α*390 variant. Notably, extensibility of *α*220 fibres compared with WT reduced similarly for uncrosslinked as for crosslinked fibrin fibres, indicating that independent of crosslinking, absence of the *α*C-region decreases fibre extensibility.

In summary, our results revealed important details about the contribution of the *α*C-region to the mechanical behaviour of fibrin fibres that is not explained by current structural models. Additionally, here we show the first direct experimental proof that strain-stiffening behaviour is present even in the absence of the *α*C region and kicks in at much larger strains than the 10–20 % expected after protofibril stretching [46]. This required a refined structural model for fibrin’s intrafibre organization, and the model we propose here fully explains the mechanical behaviour observed. Future computational modelling would be a method for testing the validity of this model.

### 4.2 Further differences between variants

While the loss of the safety latch function is connected to the loss of the flexible *α*C-region, some decrease in the high strain modulus was present already in the absence of the globular *α*C-region for crosslinked fibres. SDS-PAGE indicated reduced *α*-chain crosslinking by activated FXIII for both *α*390 and *α*220 fibrin variants. With the loss of the globular region, the interaction between the C-terminal subdomains and the *α*C-connector region is lost, an interaction which facilitates covalent crosslinking by FXIIIa [47]. Moreover, the binding site for FXIII located to residues 389–403 is largely missing in the *α*390 variant [47]. The lack of this binding site abolishes *α*-chain crosslinking, despite the fact that some of the crosslinking sites still remain present in *α*390. Thus, reduced high-strain modulus for crosslinked fibres may be due to the loss of the alpha-chain crosslinking. In agreement with this, for uncrosslinked fibres we did not see any difference in the high-strain modulus between any truncated variants. At the same time, some further nonspecific interactions [48] may still be present between the *α*C-connector regions in the *α*390 variant. Furthermore, in all variants the same connections within the protofibril backbone can form, including the *γ*-*γ* crosslinks, resulting in largely similar mechanical behaviour of all variants.

The largely similar mechanical behaviour for all different variants of individual fibrin fibres shown here is seemingly in contrast with our previous work using magnetic tweezer. These microrheology experiments showed clear and significant decrease in network strength upon the removal of the complete *α*C-region. However, importantly, *α*220 variant showed a highly abnormal, porous network structure with bundles of short, stunted fibres [26]. Consequently, the drastic decrease in strength at the microscale level reflects the disrupted network structure due to the role of the *α*C-region in the initial polymerization process, while the mechanical behaviour of the intact individual fibrin fibres remains largely similar. At the same time, the mechanical behaviour of fibres of each variant was comparable, especially up to strains of 1.5. This may suggest a role for a general mechanism, such as entropic straightening. At the fibre level, this must involve the extension of protofibrils, whereas the *α*C-region does not play a role, since low strain stiffness was similar even in the complete absence of the *α*C-region. Protofibril straightening was shown previously to play a role up to 10–20 % strain [46]. Additionally, our fluorescent microscopy images show that fibres are taut in the *X* and *Y* direction, and in cases when fibres were bent in the Z-direction we found this to be less than 1.5 μm which is less than 5 % strain. Thus, entropic straightening may play a role, but only at low (<20 %) strains, which is only a minor fraction of the full low strain region that ranges up to ~150 % strain.

In another previous study, we showed that the aspect ratio of fibrin protofibrils directly determines when fibrin assembly shifts from translationally diffusion dominated to rotationally diffusion dominated, which determines the time course of fibrin network formation [49]. We propose a similar role of geometry, but in the formation of protofibrils from fibrin monomers. The hydrodynamic radius of fibrin monomers increases when the *α*C-region undocks once fibrinogen is cleaved into fibrin, which increases the collision cross-section and helps fibrin monomers to reach one another via diffusion and consequently facilitates polymerization [26,39,40] through enhanced protofibril formation.

### 4.3 Stress relaxation

Double exponential decay during stress-relaxation has been described for various protein networks [50,51] and indicates two distinct relaxation processes. Double exponential decay has been described for fibrin fibre relaxation previously [10,38]. Our values for *τ*_1_ and *τ*_2_ are much longer (10–100 s) than reported by Hudson et al. (10–100 ms) by a huge 3 orders of magnitude [14]. However, their work investigated fibre relaxation upon release, the speed of contraction of the fibre snapping back at 0 stress, whereas here we measured relaxation following application of *additional* force and then held at constant strain. This points to different relaxation mechanisms being probed. Yet, previous studies [10,14,38] find a similar ≈10-fold difference between *τ*_1_ and *τ*_2_ as presented here. The only mechanism that appeared to have a minor but significant effect on the decay time constants was crosslinking, which is also in agreement with prior art describing a decrease in both decay constants upon crosslinking [10]. Most previous publications refrain from assigning structural elements to these relaxations and emphasize the need for testing mechanical properties on modified fibrin variants, such as the absence of the *α*C-region [38], as we performed here with truncated variants. One notable exception is the study by Hudson et al., who proposed that the entropic relaxation of the *α*C-region is the origin of the fast relaxation, while recoiling of the originally folded coiled coil and the *γ*-nodule occurs on a longer timescale [14]. This result was supported by molecular dynamic simulations. Even though their probed relaxation mechanism is different and thus relaxation times are not comparable with our values, it is interesting to consider a similar process behind fibre relaxation given the similar ≈10-fold difference between *τ*_1_ and *τ*_2_. However, we show here that both stress relaxations were present and prominent at all strains, for all truncated variants. Thus, neither of the relaxation processes can be directly assigned to the *α*C-region, or the *α*-crosslinks: even though fast relaxation only reaches its full potential when *α*-crosslinking is present, it is still present in the absence of the complete *α*C region. In our model, the structure behind any relaxation is necessarily the protofibril backbone: a fibrin fibre is a network of protofibrils and network-level relaxations often have short relaxation times [50,51], not because of unfolding events but due to reorganisations within the network. Consequently, even though our model utilizes a protofibril backbone which unfolds on longer timescales as the relaxations observed here, the network-like organisation of these protofibrils enables further mechanisms for relaxation. One such network-level relaxation mechanism is the poroelastic effect [52], where the relaxation is assigned to water outflow from within the fibre as it contracts laterally as the fibre stretches longitudinally. Fibrin fibres are indeed very porous with ~70 % water content [53] and a recent paper calculated that water expulsion is behind the vast majority (~94–98 %) of the total free energy changes during fibre elongation [15]. The stress relaxations we report here (1 − *E*_0_/*E*_max_), might also partially be due to poroelastic behaviour of fibrin fibres.

As there are no structures we can assign to the relaxations, it’s hard to address the significant increase in the relative contribution of slow relaxation when fibrin is uncrosslinked or lacks the complete *α*C-region. One possible, but hypothetical explanation might be that the increased prominence of slow relaxation is due to the decreased connectivity between protofibrils lacking *α*C-region or uncrosslinked, allowing for translation and sliding friction between adjacent protofibrils. The presence of the *α*C-region and/or crosslinks may not only bind the protofibrils, but presence of the *α*C-region may also bring protofibrils into proximity, promoting the formation of new knob-hole bonds between them. Presence of all these bonds may prevent sliding motion, increase local stiffness, and hence increase the relative proportion of fast relaxation.

### 4.4 Clinical relevance

Here we show that fibre mechanical behaviour is largely determined by protofibrils. Yet, loss of the *α*C-region reduces fibre extensibility and consequently leads to decreased fibre rupture stress and toughness. Decreased fibre toughness results in weaker clots that are more prone to rupture *in vivo* as we have previously shown in our study using an *in vivo* murine model [12]. These fragments breaking away from the blood clot will then travel on with the blood stream and cause blockages in downstream parts of the circulation such as in the lungs or the brain, leading to life-threatening conditions. In addition to the decreased fibre toughness, we have previously shown that the network-level structure also gets disrupted in the absence of the *α*C-region, especially in the absence of the flexible *α*C-domain [26]. In such a disrupted network, fibers bridging regions of clumped fibres are expected to bear higher loads. Thus, in the absence of the *α*C-region as for the *α*220 variant, not only are the fibres weaker, but they are also exposed to higher loads, which both will increase the probability of clot rupture. This is of relevance even in normal physiological conditions, as fibrinogen metabolites where the A*α*-chains are truncated at various places are present *in vivo* [54,55]. Further to this, absence of the *α*C-region makes clots more susceptible to fibrinolysis [56], weakening the structure even further. This may be of greatest importance in traumatic injuries, where a significant proportion of the *α*C-region is degraded due to fibrinogenolysis.

## 5 Conclusion

In this work we showed that low strain behaviour of individual fibrin fibres is not altered in the absence of even the complete *α*C-region and strain stiffening is still present. Absence of the *α*C-region however reduces fibre extensibility, leading to decreased fibre toughness and rupture stress. As none of the previous models of the internal structure of the fibrin fibres explain this behaviour, we propose a different approach to model of intrafibrillar structure. Our model utilizes exclusively the protofibril backbone to explain the mechanical behaviour of individual fibrin fibres. We propose that protofibrils within the fibre are frequently branching, randomly connected structures with multiple stunted ends and only a few protofibrils reaching all along the fibre length. This explains, even in the complete absence of the *α*C-region 1) the initial low stiffness of the fibres by the selective loading of protofibrils long enough to reach along the fibre 2) the strain stiffening behaviour by the increase in the number of loaded protofibrils (as so far unloaded protofibrils bind into the knobs becoming free in the initially loaded strand) and 3) the high extensibility by sliding of protofibrils relative to each other. Meanwhile the *α*C-region acts as a safety latch: it helps reinforcing the structure by pulling adjacent, unloaded protofibrils closer to the loaded ones, thus increasing the probability of the adjacent free holes to bind to the knobs becoming free due to unfolding of the loaded protofibril.

## Supplementary Material

Supplementary material associated with this article can be found, in the online version, at doi:10.1016/j.actbio.2024.10.005.

Supplement

## Figures and Tables

**Table 1:** Biomechanical parameters for truncation variants as a ratio over WT, or as a ratio of *α*220 over *α*390, before and after crosslinking with activated FXIII.

	*α* 390X/ WTX	*α* 390UX/ WTUX	*α* 220X/ WTX	*α* 220UX/ WTUX	*α* 220X/ *α* 390X	*α* 220UX/ *α* 390UX
Rupture Stress	0.794	0.8598	0.563	0.762	0.709	0.886
Toughness	0.842	0.868	0.538	0.6302	0.639	0.726
Extensibility	1.009	0.9986	0.882	0.885	0.874	0.884

X denotes crosslinked; UX denotes uncrosslinked.

## Statement of significance

The findings presented in this paper reveal critically important details about how the molecular structure of fibrin contributes to its unique mechanical properties which are essential to fulfil its function as the scaffold of blood clots. In this work we used engineered proteins with alterations in an important but highly disordered area of the molecule called *α*C-region and we provide direct evidence for the first time for how the absence of either the globular *α*C-domain, or the complete *α*C-region impacts the mechanical behaviour of individual fibrin fibres. Using these results we developed a new structural model of protofibril organisation within fibrin fibres that fully explains their strain stiffening, relatively low modulus and their high, largely variable, extensibility.
