# Supplementary material for: Fibrinogen αC-region acts as a functional safety latch: Implications for a fibrin biomechanical behaviour model
Source: Acta Biomater. Author manuscript; Available in PMC 2026 Feb 18. (PMC7618731; doi:10.1016/j.actbio.2024.10.005)
Supplement: Supplement [file EMS212469-supplement-Supplement.pdf]

Acta Biomaterialia

Supplementary Information for

Fibrinogen αC-region acts as a functional safety latch: implications for a fibrin biomechanical behaviour model

Tímea Feller[1,2]*, Helen R. McPherson[1], Simon D. Connell[2], Robert A. S. Ariëns[1]

[1] Discovery and Translational Science Department, Leeds Institute of Cardiovascular and Metabolic Medicine, University of Leeds, UK

And [2] Molecular and Nanoscale Physics Group, School of Physics, University of Leeds, UK

* Timea Feller, PhD
Email: t.feller@leeds.ac.uk

This file includes:

Supplementary text

Figures S1 to S7

Tables S1 to S5

SI References

Section S1: Fibrinogen purity and efficiency of crosslinking was tested with SDS-PAGE.

We ran an SDS-PAGE on both fibrinogen and crosslinked fibrin (FigS1/B). In WT fibrinogen the α-chain is largely intact and there is no sign of major degradation. The band of the α-chain gradually shifts to lower molecular weights for α390 and α220 fibrinogen as expected. There is a slight shift in the α- and β-chains to lower molecular weights once fibrinogen is converted to fibrin, due to the cleavage of the fibrinopeptides. In all crosslinked variants (WT, α390 and α220) the γ-chain monomer disappears and γ-dimers with higher molecular weight appear, indicating γ-chain crosslinking. For crosslinked WT, the α-chain monomer also disappears and α-polymers appear with higher molecular weight (which remain in the loading well), further indicating complete crosslinking. Most of the α-chain remains in monomer state both for α390 and α220, indicating reduced efficiency of α-chain crosslinking. This delay in alpha-crosslinking has been described before [1], and can be explained by the loss of FXIII-binding sites in the αC-region, located to residues 389-403 [2], for both truncations. These binding sites are of key importance as without them, FXIII does not bind to the α-chain and crosslinking is abolished, even though some of the crosslinking sites are still present in the flexible αC-connector region.

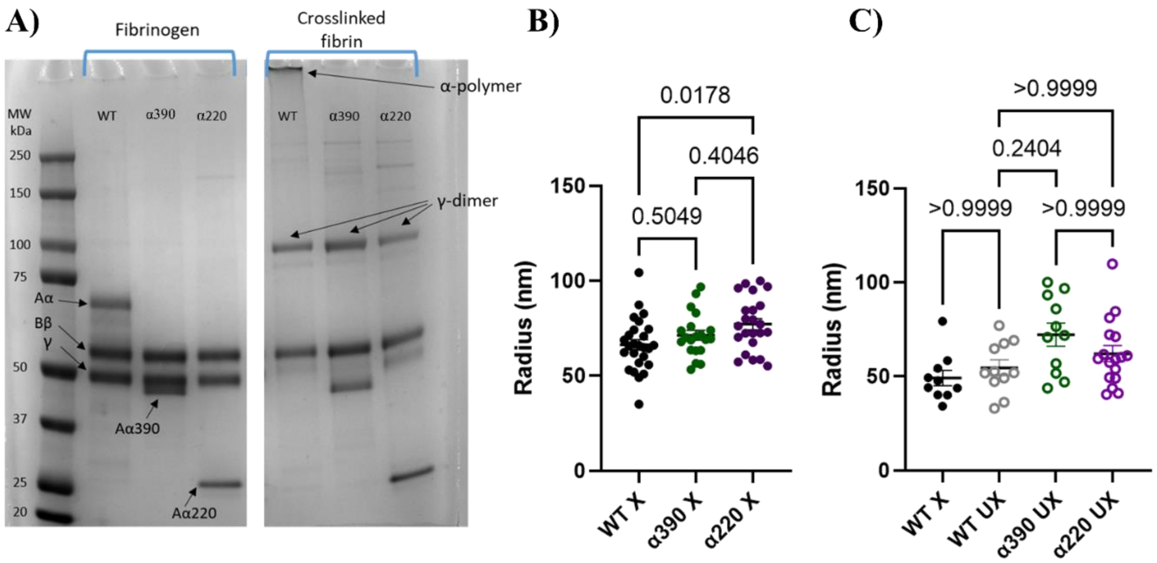

Figure S1: Sample characterisation A) Fibrinogen purity and efficiency of crosslinking was tested with SDS-PAGE. **B)** Radii of crosslinked pulled fibres. X denotes crosslinked **C)** Radii of uncrosslinked fibres with crosslinked control (WT X). UX denotes uncrosslinked.

Section S2: Testing the effect of fluorescent microbeads.

Recent evidence showed that high concentrations of microbeads alter fibrinolysis by preventing fibre cleavage [3] and cause fibres to lose their inherent tension upon lysis [4]. This is possibly due to the beads restricting access of the enzyme to fibrin by covering the surface but also opens the possibility that fibre mechanical properties are altered when labelled with microbeads. As the fluorescent microbeads cover only the surface of the fibres, we don't expect them to interfere with continuous fibre pulling, and any elongation, if present, would already be pulled out at low strains. Yet, if the beads cause elongation in the absence of lysis, that might alter fibre relaxation at incremental pulls. To test this, we labelled each variant with Alexa488, then measured fibres made with 15% labelled fibrinogen. Then we labelled the same sample with fluorescent microbeads and measured again. We found no significant differences in either the decay constants or the prominence (initial amplitude) of any of the decays.

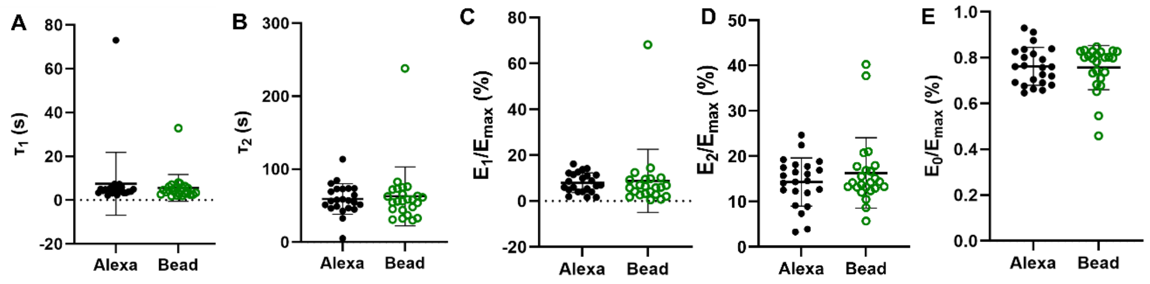

Figure S2: No significant difference was found in the fitting parameters upon fibre relaxation for Alexa488 labelled fibres or Alexa488 and fluorescent bead labelled fibres. Fitting was made using the ExpDecay function of Origin: y = y0 + A1*exp(-(x-x0)/t1) + A2*exp(-(x-x0)/t2).

Section S3: Uncrosslinked fibre pulling

In contrast with the unmeasurably low clot strength of the uncrosslinked α220 variant shown by magnetic microrheometer [1], uncrosslinked individual fibrin fibres were measurable with lateral single fibre pulling and showed largely unaltered mechanical response even in the absence of the complete αC region. We used crosslinked WT fibrinogen as control group of the uncrosslinked variants. Compared with uncrosslinked WT fibres (Fig.S3/A,B, crosslinked WT shown with black), we experienced similar changes upon crosslinking as we described previously [5]: increased High Strain Modulus (1.51-fold, 25.9 MPa vs 17.19 MPa, p=0.0396 Fig.S3/G), increased Rupture Stress (1.44-fold, 37.82 MPa vs 26.25 MPa p=0.914 Fig.S3/C) and increased Toughness (1.28-fold, 40.89 MPa vs 31.96 MPa p=0.63) (Fig.S3/D) (Table S1).

Some of the differences described for the crosslinked variants were similar in uncrosslinked variants: extensibility remained largely similar when α390 was compared to WT (0.9986-fold, p>0.9999) whereas for the α220-variant decreased ~0.88-fold compared with the WT (p=0.6491) (Fig.S3/E), which is in very good agreement with the 0.882-fold decrease of the crosslinked data. In agreement with the crosslinked results, there was also no significant difference in Low Strain Modulus for uncrosslinked fibres (all p-values are >0.9999) (Fig.S3/F). However, for uncrosslinked fibres High Strain Modulus remained largely similar in all variants and no significant decrease can be seen for the α390 and α220 variants (p>0.9999) (Fig.S3/G), unlike the decrease seen for the crosslinked variants (Fig.2/E). Likewise, tangent moduli overlap in Region II for all 3 uncrosslinked variants (Fig.S3/B), unlike the clear separation of α390 X and α220 X from WT X shown previously. Gradual removal of the αC-region resulted in gradual decrease of both rupture stress (Fig. S3/C) and toughness (Fig.S3/D), however these changes are not significant and less prominent as for the crosslinked fibres (*Table1, main text*). This milder decrease in uncrosslinked fibres is likely due to the lack of difference in stiffening and consequently in High Strain Modulus.

For the uncrosslinked dataset, rupture stress of α220 and both low and high strain moduli for WT failed the normality test (Kolmogorov-Smirnov, significance level 0.05). Consequently, nonparametric Kruskal-Wallis test was used to compare rupture stress data, and ordinary one-way Anova for comparison of extensibility and toughness data. Number of measured fibres were: WT=10, α390=11 and α220=16. Groups were measured in 2 separate parallel runs.

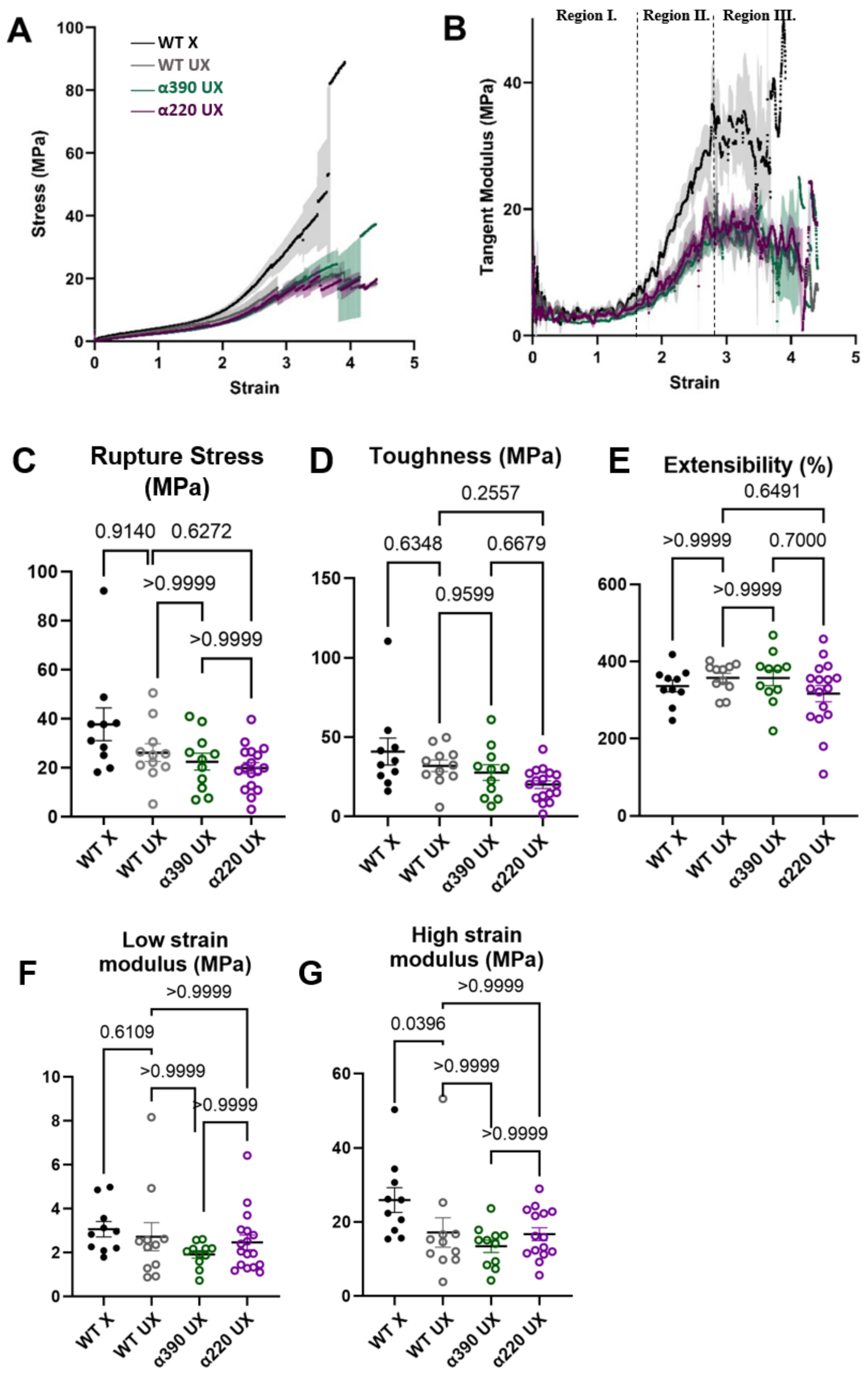

Figure S3: Uncrosslinked single fibre pulling

Row statistics ± SEM curves of all single fibre pulls for each variants (WT=10, α390=11 and α220=16). X denotes crosslinked; UX denotes uncrosslinked B) Row statistics ± SEM curves of the tangent moduli for each variants. Tangent moduli was calculated by derivation of each stress-strain curve. Adjacent averaging was used with a window of 50 data points. Resulting curves were used for row statistics. C-G) parameters were measured as shown on Fig.2/A. P-values are represented above the data.

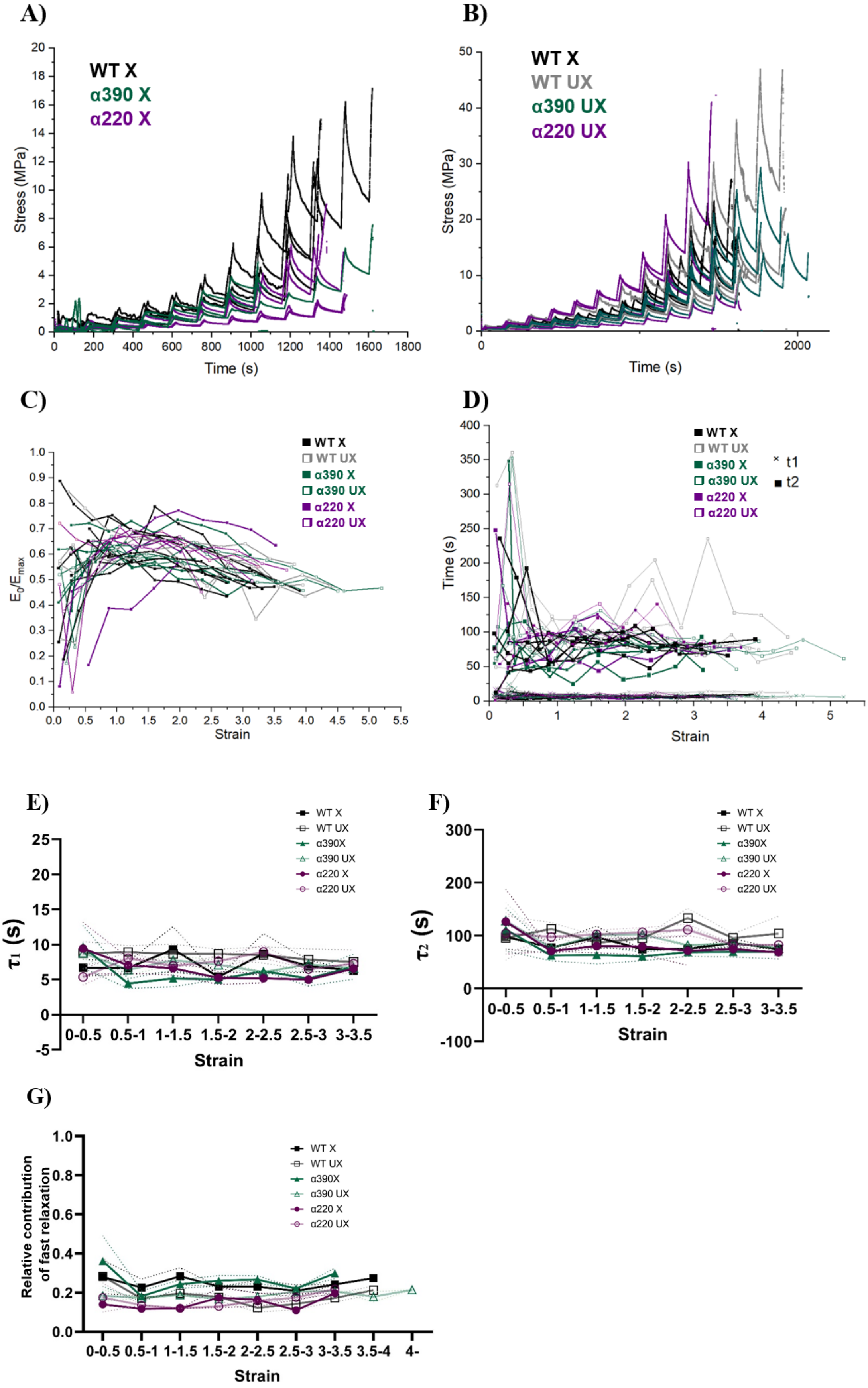

Figure S4: Incremental pulls. X denotes crosslinked; UX denotes uncrosslinked. A) stress vs time curves of all crosslinked fibres and B) all uncrosslinked fibres, with WT crosslinked control. Each curve represents one fibre. C) Elastic proportion of mechanical behaviour (E_0/E_{max}) of all fibres, crosslinked and uncrosslinked. D) All measured decay constants, τ_1 (crosses) and τ_2 (rectangles). Both crosslinked and uncrosslinked data are represented on this figure. E) Fast decay constants τ_1 grouped per strain increment of 0.5 F) Slow decay constants τ_2 grouped per strain increment of 0.5 G) Relative contribution of fast relaxation, grouped per strain increment of 0.5.

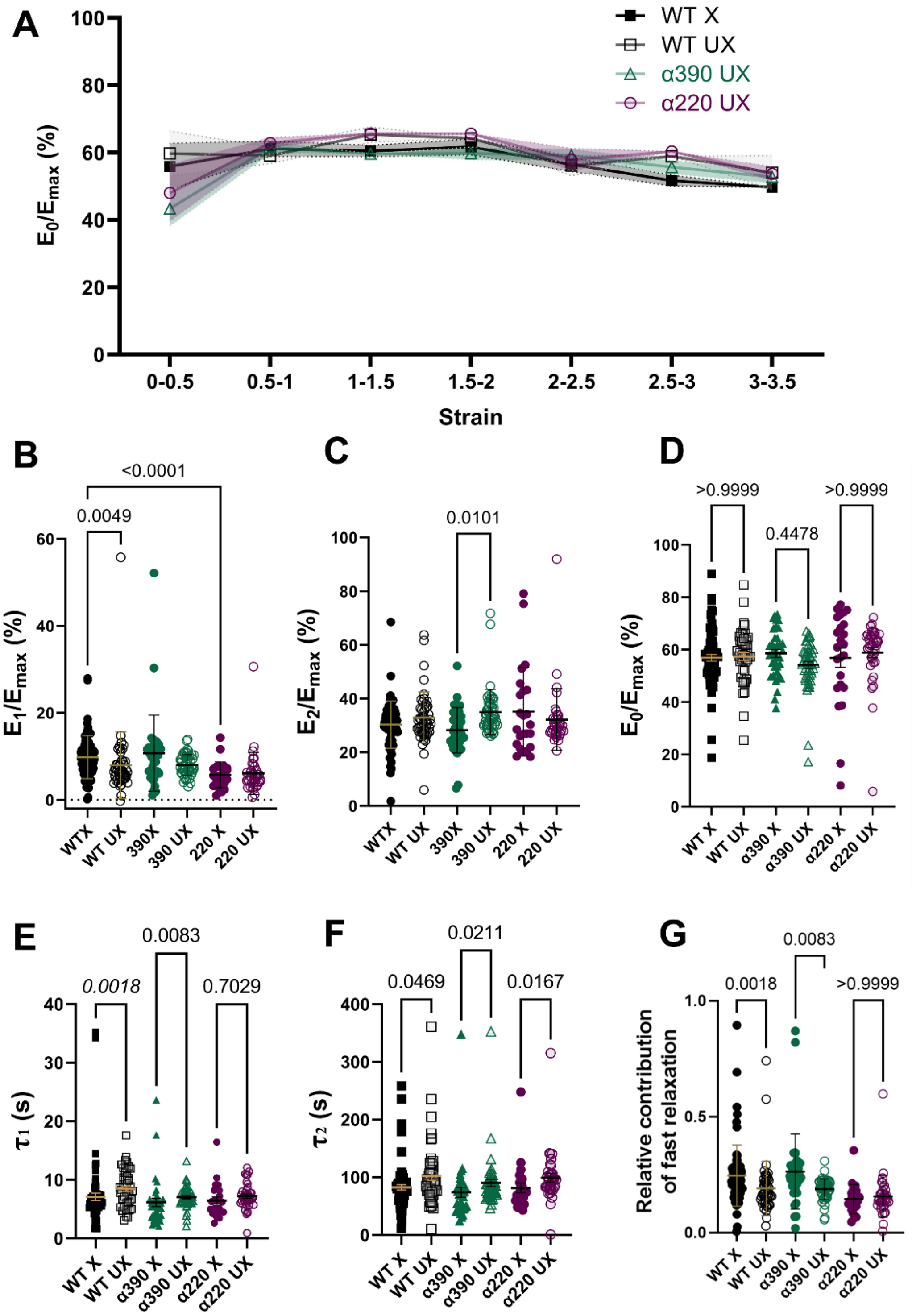

Figure S5: Incremental pulls: effect of crosslinking. A) Elastic proportion of the mechanical behaviour for uncrosslinked fibres. Crosslinked WT (WT X) was used as control. Elastic proportion of the mechanical behaviour was calculated as E_0/E_{max} (%)=$\boldsymbol{\sigma}_0/\ \boldsymbol{\sigma}_{max}$ (%). All curves represented here are average ± SEM (dashed line and shadow) of values within strain increments of 0.5. For raw data see Figure S3/C. B) Contribution of the fast relaxation to the relaxation calculated as E_1/E_{max} (%)=$\boldsymbol{\sigma}_1/\ \boldsymbol{\sigma}_{max}$ (%). C) Contribution of slow relaxation to the relaxation calculated as E_2/E_{max} (%)=$\boldsymbol{\sigma}_2/\ \boldsymbol{\sigma}_{max}$ (%). D) Elastic proportion of relaxation (E_0/E_{max} (%)) compared between crosslinked/uncrosslinked variants. No significant changes were present in the absence of crosslinking. E) Comparison of fast decay

constants τ_1 for variants, crosslinked and uncrosslinked. For each variant τ_1 increased in the absence of crosslinking, although not significantly for α220. F) Comparison of slow decay constant τ_2 for variants, crosslinked and uncrosslinked. Note the difference in Y-axis compared with τ_1. For each variant τ_2 increased significantly in the absence of crosslinking. G) Relative contribution of the fast elastic decay to all decay processes, calculated as $E_1/(E_1+E_2)$. This parameter significantly decreased in the absence of crosslinking for both the WT and α390 variants, but not for the α220 variant.

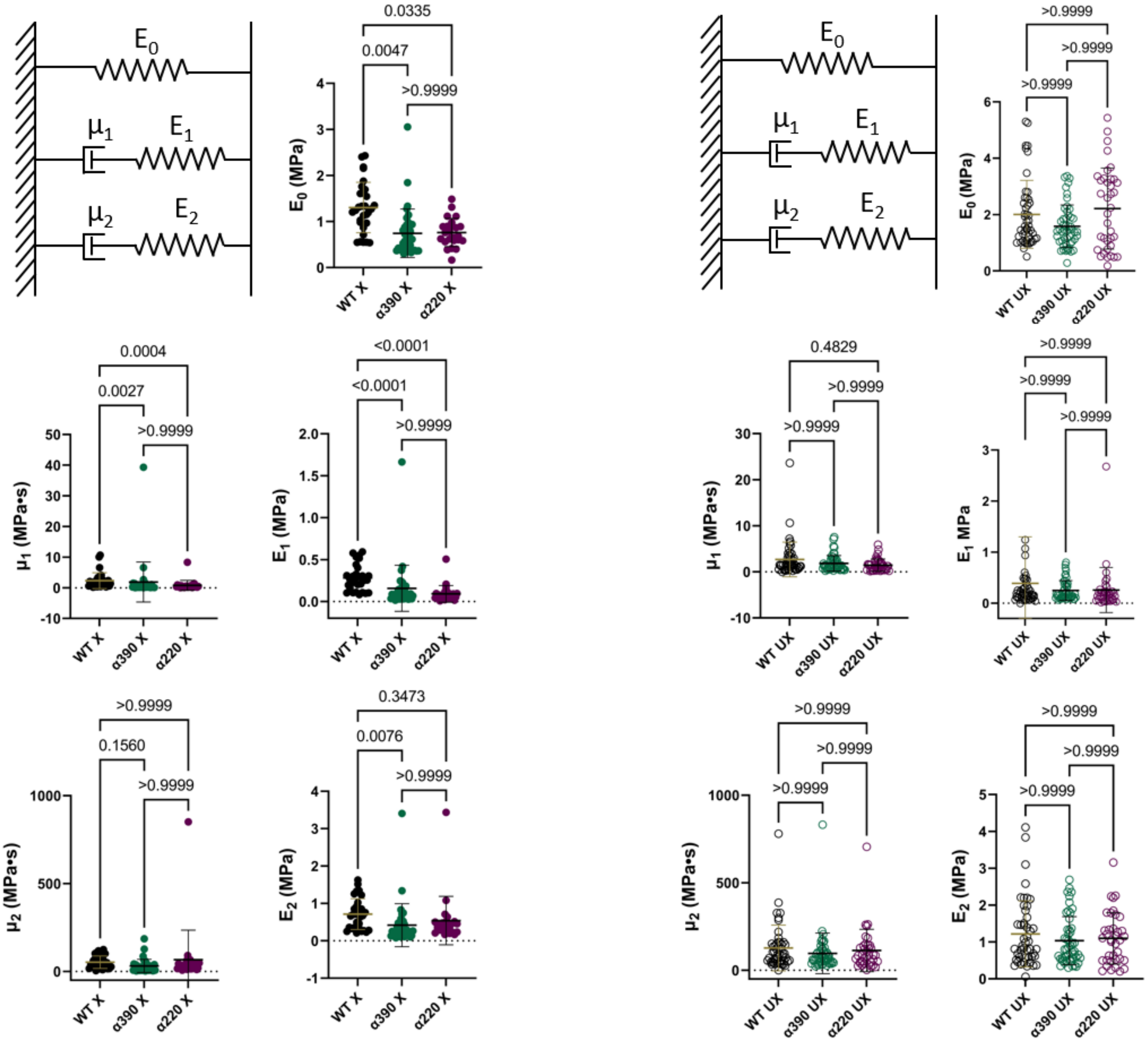

Figure S6: Kelvin model with Maxwell elements. Elastic moduli (E) were gained from the double exponential fit. Damping moduli μ was calculated utilising the Kelvin model depicted above by $\mu = E * \tau$. X denotes crosslinked; UX denotes uncrosslinked.

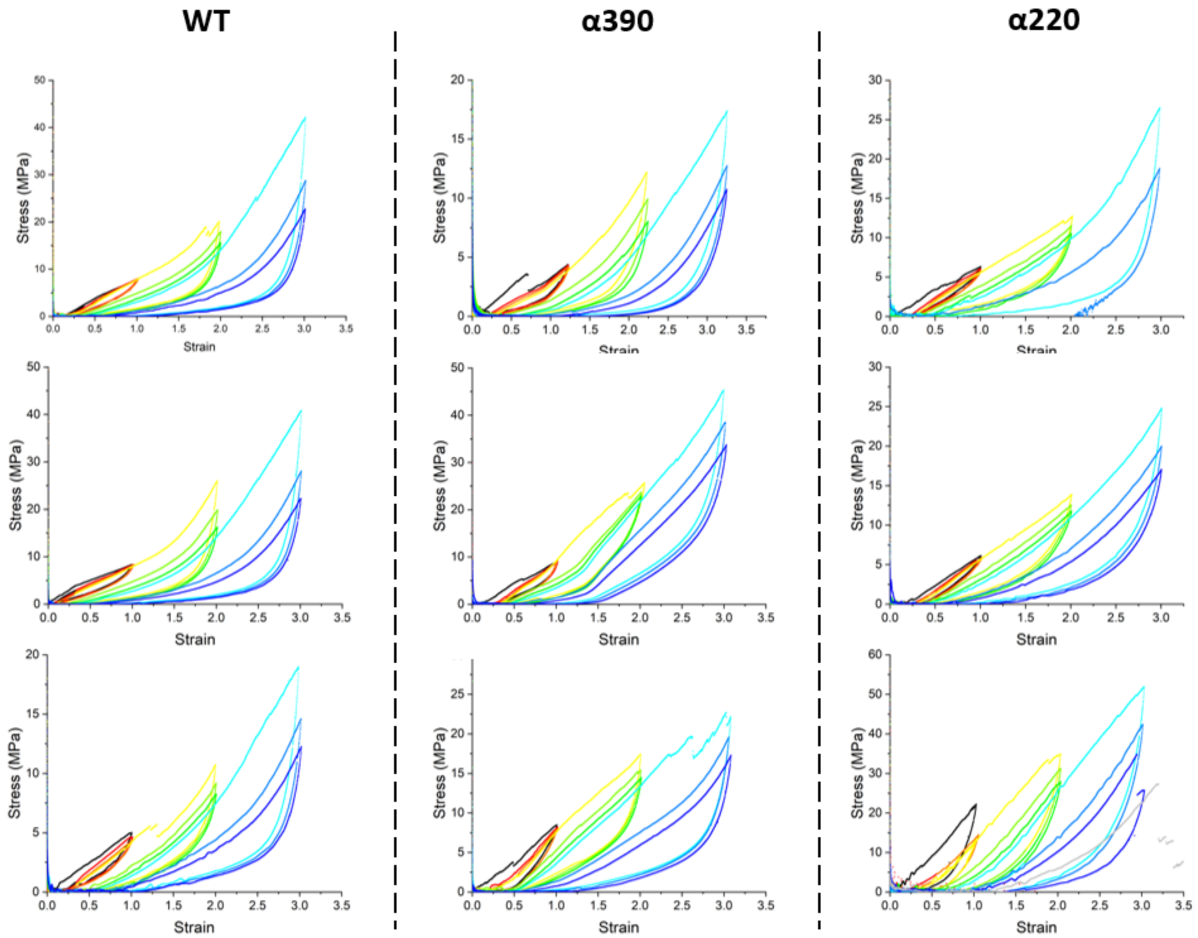

Figure S7: Cyclic fibre pulling. Three fibres of each variant were repeatedly pulled three times to strain 1 (red), then strain 2 (yellow/green) and 3 (blue), and released to strain 0 at the end of each cycle.

Table S1: Fold-wise changes in parameters upon crosslinking.

	Duval et al. PNAS 2021	In this paper:
High strain stiffness	2.2 -fold	1.51 -fold
Rupture stress	2.1 -fold	1.44 -fold
Toughness	2.0 -fold	1.28 -fold

Table S2: Mean ± SEM of all fitting parameters of stress-relaxation.

	**WTX**	**WT UX**	**390X**	**390 UX**	**220 X**	**220 UX**
**τ_1 (s),** (average ± SEM)	7.090 ± 0.6278	8.469 ± 0.4546	6.151 ± 0.6861	7.034 ± 0.2508	6.483 ± 0.5986	7.207± 0.3701
**τ_2 (s),** (average ± SEM)	85.53 ± 4.745	102.4± 8.0581	73.87 ± 8.718	90.05 ± 6.080	81.58 ± 8.410	99.33 ± 7.345
**E_0/E_{max} (%)** (average ± SEM)	56.89 ± 1.302	57.34 ± 1.470	58.57 ± 1.517	54.10 ± 1.298	56.87 ± 3.699	58.88 ± 1.918

Table S3: *Changes in decay times as ratio of crosslinked over uncrosslinked for each variants. P-values are represented in Fig.S4/C,D*

**Amount of change**	**WT X /WT UX**	**α390 X/α390 UX**	**α220 X/ α220 UX**
**τ_1 (s)**	**0.837**	0.874	0.8995
**τ_2 (s)**	0.806	0.820	0.821

Table S4: *Changes in decay times as ratio of variants over WT.*

**Amount of change**	**390X/WTX**	**220X/WTX**	**390 UX/WT UX**	**220 UX/WT UX**
**τ_1 (s)**	0.868 (p=0.9234)	0.915 (p>0.9999)	0.831 (p>0.9999)	0.851 (p>0.9999)
**τ_2 (s)**	0.864 (p=0.4463)	0.954 (p>0.9999)	0.880 (p>0.9999)	0.970 (p>0.9999)

Table S5: Changes in the contribution of fast to total relaxation [E1/(E1+E2) ratio] for each variant and with/without crosslinking

	α 220 X/WT X	α390 X/ WT X	WT UX/ WT X	α390 UX/ α390 X	α220 UX / α220 X
Change values	**0.59-fold** 14.4 % to 24.6 %	- 26.4 % to 24.6 %	**0.78-fold** 19.1 % to 24.6 %	**0.71-fold** 26.4 % to 18.7 %	- 15.7 % to 14.4 %
P value	**<0.0001**	>0.9999	**0.0018**	**0.0083**	>0.9999

X denotes crosslinked; UX denotes uncrosslinked

[1] H.R. McPherson, C. Duval, S.R. Baker, M.S. Hindle, L.T. Cheah, N.L. Asquith, M.M. Domingues, V.C. Ridger, S.D. Connell, K.M. Naseem, H. Philippou, R.A. Ajjan, R.A. Ariens, Fibrinogen alphaC-subregions critically contribute blood clot fibre growth, mechanical stability, and resistance to fibrinolysis, Elife 10 (2021).
[2] K.A. Smith, P.J. Adamson, R.J. Pease, J.M. Brown, A.J. Balmforth, P.A. Cordell, R.A. Ariens, H. Philippou, P.J. Grant, Interactions between factor XIII and the alphaC region of fibrinogen, Blood 117(12) (2011) 3460-8.
[3] N. Rimi, C.C. Helms, Labeling fibrin fibers with beads alter single fibrin fiber lysis, external clot lysis, and produce large fibrin aggregates upon lysis, Blood Coagul Fibrinolysis 33(7) (2022) 364-371.
[4] E.G. Stoll, S.J. Cone, S.R. Lynch, A.T. Fuquay, B.E. Bannish, N.E. Hudson, Fluorescent microspheres can affect in vitro fibrinolytic outcomes, PLoS One 18(4) (2023) e0284163.
[5] C. Duval, A. Baranauskas, T. Feller, M. Ali, L.T. Cheah, N.Y. Yuldasheva, S.R. Baker, H.R. McPherson, Z. Raslan, M.A. Bailey, R.M. Cubbon, S.D. Connell, R.A. Ajjan, H. Philippou, K.M. Naseem, V.C. Ridger, R.A.S. Ariens, Elimination of fibrin gamma-chain cross-linking by FXIIIa increases pulmonary embolism arising from murine inferior vena cava thrombi, Proc Natl Acad Sci U S A 118(27) (2021).
